# Genotypic and Phenotypic Characterization of Novel Sequence Types of Carbapenem-Resistant *Acinetobacter baumannii*, With Heterogeneous Resistance Determinants and Targeted Variations in Efflux Operons

**DOI:** 10.3389/fmicb.2021.738371

**Published:** 2021-12-23

**Authors:** Srinivasan Vijaya Bharathi, Manjunath Venkataramaiah, Govindan Rajamohan

**Affiliations:** Molecular Biology Division, Council of Scientific and Industrial Research-Institute of Microbial Technology, Chandigarh, India

**Keywords:** critical threat pathogen, ST195 variants, ST128 strains, antimicrobial resistance, multidrug efflux pump

## Abstract

*Acinetobacter baumannii* has emerged as one of the dominant nosocomial human pathogens associated with high morbidity and mortality globally. Increased incidences of carbapenem-resistant *A. baumannii* (CRAB) have resulted in an enormous socioeconomic burden on health-care systems. Here, we report the genotypic and phenotypic characterization of novel ST1816 and ST128 variants in *A. baumannii* strains belonging to International clone II (GC2) with capsule types KL1:OCL8 and KL3:OCL1d from India. Sequence analysis revealed the presence of diverse virulome and resistome in these clinical strains, in addition to islands, prophages, and resistance genes. The oxacillinase *bla*_*OXA–23*_detected in the genomic island also highlighted the coexistence of *bla*_*OXA–66*_/*bla*_*OXA–98*_, *bla*_*ADC73*_/*bla*_*ADC–3*_, and *bla*_*TEM–1D*_ in their mobile scaffolds, which is alarming. Together with these resistance-determining enzymes, multidrug efflux transporters also harbored substitutions, with increased expression in CRAB strains. The hotspot mutations in colistin resistance-conferring operons, PmrAB, LpxACD, and AdeRS, were additionally confirmed. Phenotype microarray analysis indicated that multidrug-resistant strains *A. baumannii* DR2 and *A. baumannii* AB067 preferred a range of antimicrobial compounds as their substrates relative to the other. To our knowledge, this is the first comprehensive report on the characterization of *A. baumannii* variants ST1816 and ST128, with different genetic makeup and genome organization. The occurrence of CRAB infections worldwide is a severe threat to available limited therapeutic options; hence, continued surveillance to monitor the emergence and dissemination of such novel ST variants in *A. baumannii* is imperative.

## Introduction

Antimicrobial resistance (AMR) is a primary public health concern across humans, plants, animals, and environmental sectors and is the recognized determinant for therapeutic failure resulting in high morbidity and mortality worldwide. Albeit the global antibiotic pipeline as of December 2020 enlists the development of 43 promising drugs, the alarming upsurge in AMR infections still demands the search for novel classes of antibiotics^1^.

In May 2015, the World Health Organization (WHO) published its national action plan, emphasizing global health governance by improving awareness, strengthening surveillance, and infection control (Mendelson and Matsoso, 2014, 2015; Shallcross and Davies, 2014; Shallcross et al., 2015). In contrast to its previous programs, the WHO’s overall goal behind prioritizing pathogens as critical, high, and medium was to facilitate funding and promote research and development partnerships to discover novel anti-infective (WHO, 2017). India too has enormous distress due to multidrug-resistant infections; hence, to enhance the epidemiological monitoring and evaluate the extent of AMR burden, the Indian Government initiated the research and surveillance program [Indian Council of Medical Research—Antimicrobial Resistance Surveillance and Research Network (ICMR-AMRSN)] in 2013 (Laxminarayan et al., 2013; Kaur et al., 2019; Veeraraghavan and Walia, 2019). The prime mandate of ICMR-AMRSN is to establish a multicenter network and standardize protocols for strain identification, genetic typing, antibiotic susceptibilities, deciphering varied molecular mechanisms behind resistance, and develop a data collection and management system (Ghafur et al., 2013; Taneja and Sharma, 2019; Walia et al., 2019). Together with 143 countries that developed their national action plans, on April 19, 2017, India published its first AMR combat plan with equivalent objectives (Kakkar et al., 2018; Muthuirulandi Sethuvel et al., 2019; Ranjalkar and Chandy, 2019). The WHO Indian office and Department of Biotechnology published the first Indian pathogen priority list recently to guide future novel antimicrobial interventions against major clinically significant bacteria.

The notorious antibiotic-resistant *Acinetobacter baumannii* is the “top critical threat” pathogen in the list among *Enterococcus faecium*, *Staphylococcus aureus*, *Klebsiella pneumoniae*, *A. baumannii*, *Pseudomonas aeruginosa*, and *Enterobacter* spp. organisms attributed to high mortality, huge clinical-socioeconomic burden, and international circulation of multidrug-resistant clonal complexes with limited availability of therapeutic options (Falagas and Rafailidis, 2007; Wong et al., 2017; Chen et al., 2018; Harding et al., 2018). According to the recent ICMR-AMR data, of the total 107,387 isolates studied during 2019, the relative distribution of *A. baumannii* remained the highest at 23.6% in Indian intensive care units from different locations (Vijayakumar et al., 2020a,b).

*Acinetobacter* is a strictly aerobic, Gram-negative coccobacillus rod, a motile, catalase-positive, oxidase-negative, non-fastidious bacterium from the family Moraxellaceae and phylum proteobacteria (Lee et al., 2017). Members of this genus are frequently found in water, soil, sewage effluent, food items, and human skin and account for up to 10% of all Gram-negative nosocomial infections (Fournier and Richet, 2006; Giamarellou et al., 2008). Amidst 60 members of this genus, such as *Acinetobacter calcoaceticus*, *Acinetobacter pittii*, *Acinetobacter nosocomialis*, *Acinetobacter seifertii*, and *Acinetobacter lactucae*, the most frequently encountered and characterized is *A. baumannii* (Larson, 1984). This hard-to-treat microbial agent invades immunosuppressed hosts and is responsible for causing skin, soft tissue, and abdominal infections to complicated ones such as meningitis, pneumonia, bloodstream infections, acute respiratory syndrome, and urinary tract infections (Bergogne-Berezin and Towner, 1996; Peleg et al., 2008; McConnell et al., 2013). *A. baumannii* is a troublesome pathogen in humans; its genome contains an arsenal of virulence factors responsible for causing the vast array of nosocomial infections (Dijkshoorn et al., 2007; Weber et al., 2015).

Bacterial adhesions, hemolysins, and fimbriae help pathogen evade the host immune response, and crucial enzyme phospholipases A, D, and C with hemolytic activity regulated by ferric uptake regulator are considered essential for *in vivo* pathogenesis, epithelial cell invasion, and serum resistance (Singh et al., 2018; Geisinger et al., 2019). Capsular polysaccharides are surface exposed layers that exhibit resistance to cationic antimicrobial peptides promoting *in vivo* survival (Lees-Miller et al., 2013; Schmid et al., 2015). Talyansky et al. (2021) have recently reported that a single disruption in the capsule synthesis loci, glycosyltransferase gene, *gtr6* renders *A. baumannii* highly virulent, clearly demonstrating the critical role of capsule sugar composition in the virulence potential of the bacteria and other host immunity responses.

Outer membrane vesicles secreted by *A. baumannii* are crucial for biofilm formation, gene transfer, quorum sensing, inhibition of phagosomal maturation in macrophages, and virulence gene transportation (Chatterjee et al., 2017; Kim et al., 2019). The bacilli contain several secretion systems (types I, II, and IV), and expression of the type VI system augments bacterial fitness and its survival in host and abiotic surfaces (Repizo et al., 2015; Harding et al., 2016; Wang et al., 2018; Elhosseiny et al., 2019; Lopez et al., 2020). Together with a zinc acquisition system and a manganese transporter, *A. baumannii* also expresses proteins to meet its need for iron, including the direct uptake system and high-affinity chelators, called siderophores (Proschak et al., 2013; Penwell et al., 2015; Bohac et al., 2019). The scavenger acinetobactin has a crucial role in virulence, helps bacteria survive in a stressed condition, and induces host cell damage and death, whereas fimsbactins and baumannoferrin remain completely dispensable (Penwell et al., 2015). Besides nutrient availability, macromolecular secretions, pili expression, environmental signals, the heat-modifiable protein OmpA, and biofilm-related protein BamA all together have a significant role in modulating biofilm formation and adhesion in *A. baumannii* (Singh et al., 2014, 2017; Lin et al., 2015; Nie et al., 2020). The virulence factors exposed on cell-envelope include Omp33–36, Omp22, CarO, OmpW, LptD, and OprD, which protect cells against physiological response, alter cell envelope integrity to being assailed by harsh environmental conditions, as well as exposure to treatment options, such as cephalosporins and carbapenems (Catel-Ferreira et al., 2012, 2016; Bojkovic et al., 2015; Abellón-Ruiz et al., 2018; Guo et al., 2018; Khorsi et al., 2018).

Several reports on the increased incidences and propagation of carbapenem-resistant *A. baumannii* (CRAB) across continents in the last few years have received physicians’ attention. Carbapenem resistance is a severe problem affecting Africa’s health-care system, where the occurrences have surged from <1 to 60% among Gram-negative bacteria (Anane et al., 2020). In a recent study carried out in Nepal’s tertiary care hospital, of the 177 *A. baumannii* studied, nearly 91% of them exhibited carbapenem resistance (Devkota et al., 2020). Grisold et al. (2021) recently reported that among the 114 *A. baumannii* isolates characterized, 97.4 and 98.2% were resistant to imipenem and meropenem, whereas 88.6% exhibited multidrug resistance. As per the ICMR-AMRSN data, 45% of hospital-acquired infections are due to *Acinetobacter* spp., with higher non-susceptibilities to imipenem (82.2%) followed by meropenem (78.3%), cefepime, and ceftazidime (87%) (Muthuirulandi Sethuvel et al., 2019). The first report from the cohort study of ICMR-AMRSN discusses that 75 *A. baumannii* non-repetitive isolates collected during 2015–2017 from five different nodal centers in India exhibited 100% resistance to meropenem and imipenem (Vijayakumar et al., 2020b). Its sequel report involving 763 non-duplicate isolates from eight different study centers highlighted that all the tested isolates were resistant to both imipenem and meropenem and harbored relevant resistance determinants to express the phenotype (Vijayakumar et al., 2020a).

Diverse acquired and intrinsic molecular mechanisms mediate extreme carbapenem resistance in *A. baumannii*. Dissemination of the first OXA enzyme *bla*_*OXA–23–like*_, and other *bla*_*OXA–143–like*_, *bla*_*OXA–51–like*_, *bla*_*OXA–24/40–like*_, *bla*_*OXA–235–like*_, and *bla*_*OXA–58–like*_, with carbapenemase activity, is reported from different countries, as a part of a mobile genetic element or transposon (Poirel et al., 2010). Another circulating resistance determinant known to confer carbapenem resistance in *A. baumannii* is the zinc-dependent *bla*NDM-1, primarily reported from an Indian isolate as a part of a 180-Kb mega-plasmid (Karthikeyan et al., 2010). Apart from these enzyme-mediated mechanisms, CRAB strains also exhibit loss of channel-forming porins, namely 29-kDa CarO and 43-kDa OprD, leading to defective membrane permeability (Siroy et al., 2005; Catel-Ferreira et al., 2012). Gehrlein et al. (1991) demonstrated that CRAB produced a 24-kDa PBP, and another study illustrated the reduced expression of 73.2-kDa PBP. Few studies elucidated the role of overexpressed resistance nodulation division (RND) efflux systems, AdeIJK, AdeABC, and AdeFGH, combined with carbapenemase expression toward carbapenem non-susceptibility (Abdi et al., 2020).

Specific clonal complexes of CRAB have mainly been responsible for outbreaks in hospitals from different countries, including the United States, South America, Canada, Europe, Australia, and Asia. Most of the CRAB strains belonged to the global clone CC2 and sequence type (ST)2 (Diancourt et al., 2010; Zarrilli et al., 2013).

One of the aims of our research design is to periodically evaluate the changing trends of antibiogram and identify the dominant resistance determinant in *A. baumannii* isolates from India. Drug susceptibility data helped us identify three distinct *A. baumannii* clinical strains DR1, DR2, and AB067, which belong to novel STs with different susceptibilities toward carbapenems, aminoglycosides, quinolones, and biocides. The objective of this study was to perform genome sequencing of these selected *A. baumannii* strains, followed by comparative genome sequence and phenotypic analysis to highlight the similarities/differences in the genetic makeup of these novel STs and characterize them to enlist the complete antibiotic resistome for their extreme renitent behavior.

This report comprehensively describes the genotypic and phenotypic characterization of novel *A. baumannii* variants, ST2 (ST1816) and ST49 (ST128) that belongs to International clone –II isolated from India for the first time.

## Materials and Methods

### Bacterial Isolate and Identification

The *A. baumannii* DR1, DR2, and AB067 strain used in this study was obtained from a biological sample during our longitudinal studies, which was to deduce the emerging traits of AMR mechanisms. The isolate was characterized using 16S ribosomal DNA sequencing and matrix-assisted laser desorption/ionization-time of flight assay, with matrix-assisted laser desorption/ionization biotyper software (Bruker Daltonics, Germany), and confirmed to be *A. baumannii*. The susceptibility profile for different antibiotics, dyes, detergents, and hospital-based disinfectants was evaluated following standard Clinical and Laboratory Standards Institute (CLSI) guidelines for different antimicrobials (Clinical and Laboratory Standards Institute, 2014). The institutional bio-safety committee has approved this study.

### Draft Genome Sequencing, Its Assembly, and Annotation

The chromosomal DNA from these clinical strains *A. baumannii* DR1, DR2, and AB067 were isolated using the ZR bacterial/fungal miniprep DNA kit (from Zyme Research Corporation, Irvine, CA, United States), and the obtained DNA was checked for purity and quantified using the NanoDrop (Thermo Scientific). The genomic DNA library was prepared using the Illumina sequencing library preparation in Genotypic Technology (P) Ltd., Bengaluru, and genome sequencing facility using the NEXTFlex DNA library procedure as described in the DNA sample preparation manual NEXTFlex (Illumina, Inc., San Diego, CA, United States). Using Illumina NextSeq 500 Paired-end sequencing technology, the size distribution of the sequencing library and quantification were performed. The quality of paired-end raw reads was determined using the Fast QC^2^ and further analyzed by Genotypic Technology’s perl language for adapters and low resolved bases, and the shortening started from 3’ end. *De novo* assembly of Illumina NextSeq adapter free and error correction of data was performed using SPAdes assembler (Bankevich et al., 2012).

Scaffolding of the assembled contigs was carried out using SSPACE and removal of extra N that got inserted during scaffolding using GapCloser. The scaffold sequence annotation was carried out using the National Center for Biotechnology Information (NCBI) pipeline and RAST server (Aziz et al., 2008). The pathway analysis was done using KASS and MISA^3^, and the draft genome was compared with the nearest reference genome using CONTIGuator.

The genome data of the Indian *A. baumannii* isolates obtained in this study were primarily compared with *A. baumannii* strain ACICU (accession number NZ_CP031380.1). However, to get a clear picture of the genetic disparity, further comparisons were made with other sequenced *A. baumannii* strains isolated from different niches available in the NCBI genome database, to name a few *A. baumannii* strain AC12 (accession number CP007549.3), *A. baumannii* strain 29 (accession number NZ_CP007535.2), *A. baumannii* strain 30 (accession number CP007577.1), *A. baumannii* strain TYTH-1 (accession number NC_018706.1), and *A. baumannii* strain MDR-TJ (accession number NC_017847.1).

The BLAST-based tabulation of pair-wise average nucleotide identity (ANI) scores was calculated using JSpecies for the genome sequence similarity analysis. The widespread use of ANI scores for analysis by researchers proves its acceptance as the next-level gold standard for species identification.

The core genome single-nucleotide polymorphism-based alignment of selected *A. baumannii* strains was generated with the online tool REALPHY^4^ using *A. baumannii* ACICU as the reference genome. The unrooted phylogenomic tree was inferred by the maximum likelihood method using a generalized time-reversible model, analyzed and visualized using an interactive tree of life^5^.

The prophage regions were identified using PHASTER^6^. Genomic islands were identified using Island viewer. The AMR genes present in the *A. baumannii* strains DR1, DR2, and AB067 were identified from available databases that consist of different antibiotic resistance determinants such as CARD^7^, PATRIC, ResFinder. Sequence analysis to identify mutations was performed using the *A. baumannii* strain ATCC 17978 as a template (CP000521).

### Antibiotic Susceptibility Testing and Growth Inhibition Assays

The *A. baumannii* strains DR1, DR2, and AB067 strains were assessed for antibiotic susceptibility using Kirby Bauer disk diffusion assay; antibiotics disks (in μg/ml) from HiMedia Laboratories, India, used were amikacin, ampicillin, azithromycin, ceftazidime-avibactam, ceftazidime, ceftriaxone, chloramphenicol, ciprofloxacin, colistin, co-trimoxazole (Sulpha/Trimethoprim), doripenem, doxycycline, ertapenem, erythromycin, gentamicin, imipenem, kanamycin, meropenem, methicillin, minocycline, oxacillin, streptomycin, tetracycline, and tigecycline, and the data were tabulated. The minimum inhibitory concentration (MIC) was deduced using *E*-test, and the CLSI quality control strain *Escherichia coli* ATCC 25922 was used as experimental controls. The MIC for polymyxin B was determined by the LB broth method. The phenotypic assessment for active efflux activity was examined by determining the MICs using E-strips in the presence and absence of efflux pump inhibitors carbonyl cyanide 3-chlorophenylhydrazone (CCCP) and reserpine. Briefly, an efflux inhibitor was added to the LB agar plate at the final concentration of 5 μg/ml. The growth inhibition assay, biofilm formation testing, and MIC for biocides were determined as described before (Srinivasan et al., 2015; Srinivasan and Rajamohan, 2020a).

### RNA Isolation and Real-Time Reverse Transcription-Polymerase Chain Reaction

Total RNA was isolated from the stationary phase of growth and purified as described previously (Srinivasan et al., 2015). DNase I-treated RNA was used for complementary DNA synthesis using superscript III reverse transcriptase (Invitrogen). The generated complementary DNA was used as a template for monitoring gene expression levels by real-time reverse transcription-polymerase chain reaction with SYBR green supermix (Bio-Rad) in Mastercycler ep realplex (Eppendorf). Melt curve analyses were performed to ensure desired amplicon, and transcriptional variations were calculated using *A. baumannii* DR1 strain as a control. Experiments were performed in duplicates with *rpoB* as an internal control.

### Phenotype Microarray Testing

The phenomic analysis of *A. baumannii* strains DR1, DR2, and AB067 were assayed using Biolog Phenotype MicroArray (PM) system as per manufacturer’s protocol to identify the sensitivities toward different stress (PM9 and PM10) and antimicrobial compounds (PM11–PM20). Briefly, the strains were streaked on LB agar plates overnight at 37°C, and the single colony cell suspension was made with 85% transmittance in Biolog IF-0 inoculation fluid (Biolog, Inc). Subsequently diluted in Biolog fluid containing dye A (Biolog, Inc.), further 100 μl of diluted cell suspension was added to each well of the Biolog microplates and incubated in Omnilog automatic plate reader (Biolog, Inc.) for 48 h at 37°C. Color formation from the redox-active tetrazolium-based dye (Biolog dye A) from colorless to formazan (violet) was monitored every 15 min. Data obtained from the respiration rate of *A. baumannii* strains were individually overlaid against the control (*A. baumannii* DR1) using the OmniLog File Management/Kinetic Analysis software v1.20.02 and analyzed using OmniLog Parametric Analysis software v1.20.02 (Biolog, Inc.). The growth curve height greater than 101 Omnilog units was considered as a positive phenotype, and higher values due to coloration of certain compounds were excluded from the analysis.

### Statistical Analysis

Data were statistically analyzed by analysis of variance tests using the GraphPad Prism (GraphPad Software, San Diego, CA, United States). The *p*-value < 0.05 was considered statistically significant.

## Results and Discussion

### Genotypic Characterization of *Acinetobacter baumannii* Strains DR1, DR2, and AB067

#### Genome Features

The draft genome reads of *A. baumannii* strain DR1 assembled (GCA_002265555.1) into a single chromosome of size 3,942,977 Mb, having 38.8% G + C content; the number of contigs being 37, with N50 272,246 bp, L50 as 6 (GenBank accession number NOXP01). Genome sequence analysis of *A. baumannii* strain DR1 revealed the presence of 3,845 genes (total), 3,769 CDS (total), 3,721 coding genes, 76 RNA genes, 64 tRNAs, and 48 pseudogenes.

In contrast, the genome reads of *A. baumannii* strain DR2 assembled (GCA_002265675.1) into a single chromosome of size 3,897,527 Mb, having 39.0% G + C content; the number of contigs being 102, with N50 157,362 bp, L50 as 10 (Genbank accession number NOXO01), and *A. baumannii* strain DR2 consisted of 3,810 genes (total), 3,737 CDS (total), 3,668 coding genes, 73 RNA genes, 63 tRNAs, and 69 pseudogenes.

**FIGURE 1:**
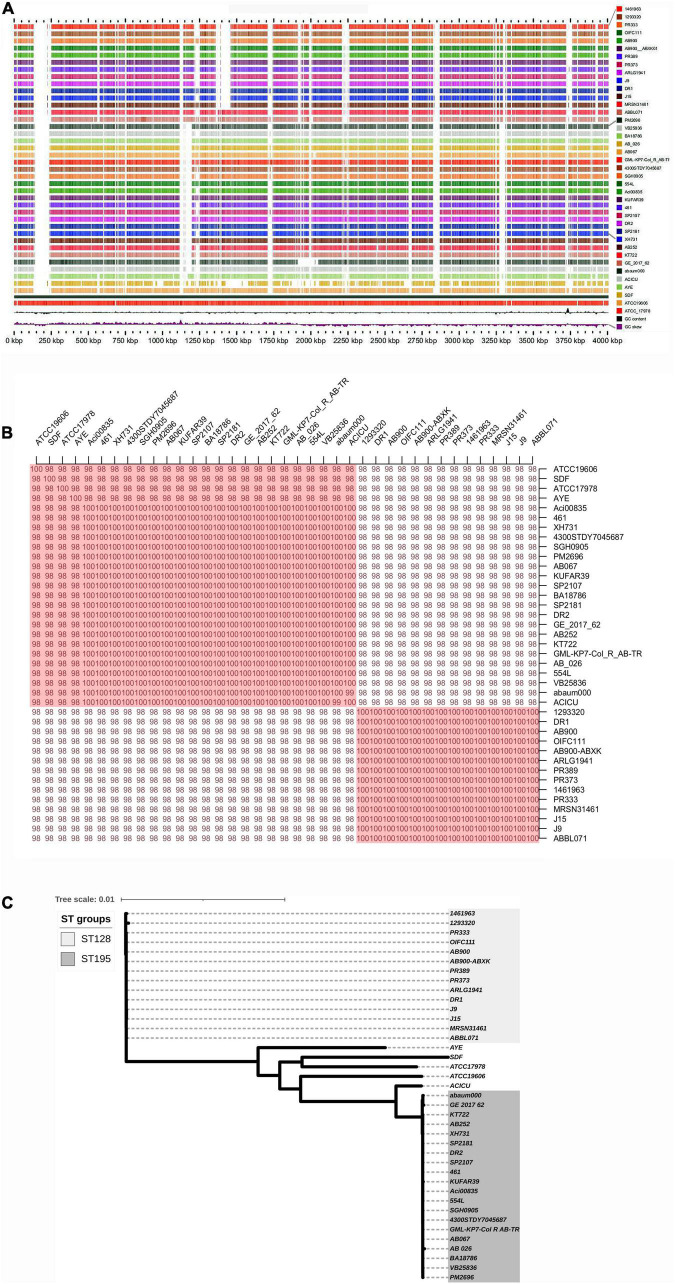
**(A)** Linear genome map of *A. baumannii* strains DR1, DR2, and AB067 with other completely sequenced strains. Different strains used are *A. baumannii* strain 1461963 (JEWQ01), 1293320 (JFEE01), PR333 (NGDW01), OIFC111 (AMFY01), AB900 (ABXK01), AB900 (JAAVKC01), PR389 (NGEW01), PR373 (NGDA01), ARLG1941 (NGIN01), J9 (CP041587), DR1 (NOXP01), J15 (VKGL01), MRSN31461 (VHFT01), ABBL071 (LLGB01), PM2696 (JAAGSK01), VB25836 (RHLW01), BA18786 (JAAEGP01), AB_026 (RIAH01), AB067 (LZOC01), GML_KP7_Col_R_AB_TR (QHHE01), 4300STDY7045687 (UFIZ01), SGH0905 (PYEH01), 554L (NKXQ01), Aci00835 (VAFQ01), KUFAR39 (NEPK01), 461 (LCTE01), SP2107 (JAAGSY01), DR2 (NOXO01), SP2181 (JAAGTC01), XH731 (CP021321), AB252 (LXNE01), KT722 (RXIN01), GE_2017_62 (WIHB01), abaum000 (UWVS01), ACICU (CP031380), AYE (CU459141), SDF (CU468230), ATCC19606 (CZWC01), and ATCC_17978 (CP000521). Accession numbers are mentioned in parentheses for each genome. **(B)** Average nucleotide identity (ANI) analysis of *A. baumannii* strains DR1, DR2, and AB067 with other sequenced strains; values clearly indicate nucleotide level genome similarity in MDR strains used in this study belong to international clone 2. **(C)** Phylogenomic tree inferred based on single-nucleotide polymorphisms (SNP) in *A. baumannii* strains using maximum likelihood method. Unrooted phylogenomic tree was analyzed, and strains *A. baumannii* DR1 belong to ST128 (ST49) group, whereas both *A. baumannii* strains DR2 and AB067 that belonged to ST1816 (ST195) were distinctly clustered.

The genome sequence reads of *A. baumannii* strain AB067 assembled (GCA_001909135.1) into a single chromosome of size 3,872,679 Mb, having 39% G + C content; the number of contigs being 60, with N50 148,676 bp, L50 as 10 (Genbank accession number LZOC01). In contrast, *A. baumannii* strain AB067 displayed the presence of 3,711 genes (total), 3,638 CDS (total), 3,568 coding genes, 73 RNA genes, 61 tRNAs, and 70 pseudogenes. The linear genome maps of the clinical strains are shown in Figure 1A.

The *A. baumannii* strain DR1 belongs to ST49 (ST128), which genomically relates closely to *A. baumannii* strain PR333 (NGDW01), J9 (NZ_CP041587), PR373 (NGDA01), AB900 (JAAVKC), and 1461963 (JEWQ01) as they assemble together in the same cluster as per the ANI scores and phylogeny analysis (Figures 1B,C).

Similarly, *A. baumannii* strain DR2 and AB067 clustered under ST195 (ST1816), closely resembles other members in the clade, namely ACICU (NZ_CP031380), SP2181 (JAAGTC), BA18786 (JAAEGP), KUFAR39 (NEPK01), PM2696 (JAAGSK), and XH731 (NZ_CP021321) (Figures 1B,C).

As per the RAST server, the functional composition of *A. baumannii* DR1, DR2, and AB067 genomes revealed several subsystems (458, 457) designated in the chromosome representing approximately 49% of classified sequences (51% unassigned).

The coding gene distribution (in percentage) for different functions in the genomes of *A. baumannii* strains DR1, DR2, and AB067 are as follows: for amino acids and derivatives (17.8, 15.4, and 15.6%), protein metabolism (12.1, 8.51, and 8.51%), carbohydrates (10.9, 10.7, and 10.7%), stress response (3.67, 4.25, and 4.25%), iron acquisition and metabolism (1.26, 1.02, and 1.07%), regulation and cell signaling (2.09, 2.97, and 3.00%), phages, transposable elements (0.95, 1.064, and 0.75%), and membrane transport (5.13, 3.93, and 3.97%) (Supplementary Figures 1A,B).

The *A. baumannii* strain DR1 displayed the presence of prophages of different sizes (34,131, 55,217, 77,242, 126,717, 49,042, and 30,064 bp) in its genome sequence, with a GC content ranging between 37.71 and 39.66% (Supplementary Figure 2A); The drug-resistant *A. baumannii* strain DR2 also showed the presence of prophages of different sizes (52,055, 87,316, 8,894, and 70,664 bp) in its genome sequence, with a GC content ranging between 36.92 and 41.19% (Supplementary Figure 2B), and *A. baumannii* strain AB067 had prophages of different sizes (64,203, 84,818, 51,529, and 65,238 bp; with GC content 36.92 to 40.61%), with the additional presence of type VI secretion system in these clusters (Supplementary Figure 2C).

### Virulence Genes Present in *Acinetobacter baumannii* Strains DR1, DR2, and AB067

#### Virulence Genes: Outer Membrane Proteins

The major virulence factor OmpA (Omp38) performs a dual function of evasion and invasion to establish chronic infection and induce host cell apoptosis by augmenting biofilm formation, serum resistance, and evading complement attack. The structural details highlight the presence of an eight-stranded β*-*barrel N-terminal domain (amino acid positions: 1–172) with four surface-exposed extended loops, the linker region (173–187) and the globular C-terminal domain (188–221), and the NCBI domain search confirmed the conserved presence of this transmembrane less soluble OmpA porin in these MDR strains with >92% identity to A1S_2840 from *A. baumannii* ATCC 17978. The presence of essential residues known to bind diaminopimelate of peptidoglycan for maintaining cell membrane integrity Asp271 and Arg286, together with arginine/lysine-rich nuclear localization signal KTKEGRAMNRR (320–330), responsible for its translocation into the nucleus to instigate host cell degradation, was also found intact in these 36-kDa porin homologs found located in NODE_7_length (CHQ89_10425), NODE_16_length (CHQ90_12710), and scaffold 13 (A8A08_03680) of *A. baumannii* strains DR1, DR2, and AB067, respectively.

**FIGURE 2:**
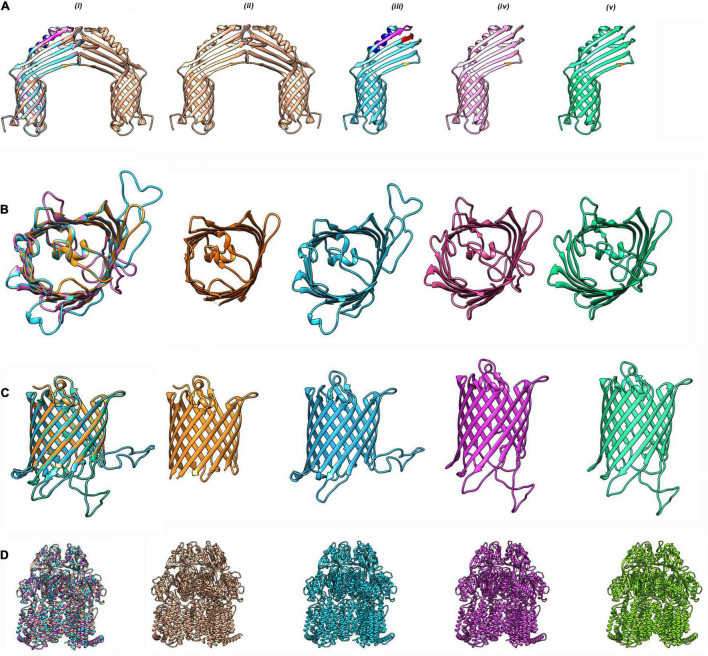
Homology modeling of antibiotic resistance proteins in CRAB strains. **(A)** CarO from *A. baumannii* strains DR1, DR2, and AB067 strains were modeled using PDB 4fuv.1 as template in SWISS model (https://swissmodel.expasy.org) and visualized with Chimera 1.15 tool. **(i)** Superimposition of modeled monomeric CarO encoded by DR1, DR2, and AB067 strains were aligned with homo-dimeric CarO (PDB 4fuv.1). Homodimeric CarO (PDB 4fuv.1) **(ii)**, monomeric CarO from DR1 **(iii)**, DR2 **(iv)**, and AB067 **(v)** are displayed as modeled proteins. Mutations are highlighted in different colors. **(B)** Top view of overlapped monomeric Omp33–36 from DR1, DR2, and AB067 strains with *A. baumannii* Omp33 (PDB 6gie.1) as template **(i)**, panel **(ii)** *A. baumannii* Omp33 (PDB 6gie.1), **(iii–v)** corresponds to homology modeled monomeric Omp33–36 proteins of DR1, DR2, and AB967, respectively. **(C)** Side view of merged Omp33–36 modeled proteins from DR1, DR2, and AB067 strains (**i–v** same as **B**). Different strains displayed difference in loop regions. **(D)** Modeled AdeB multidrug resistance efflux pump from DR1, DR2, and AB067 strains were aligned with target template PDB 7kge.1 **(i)** and *A. baumannii* trimeric AdeB (PDB:7kge.1) **(ii)**, modeled AdeB from DR1 **(iii)**, DR2 **(iv)**, and AB067 **(v)** strains, respectively.

Similarly, CarO, another porin known to have a role in carbapenem resistance, was also found to be structurally distinct in ST49; as compared with ST195, it will be of interest to investigate its influence on impacting membrane permeability, leading to the altered drug resistance phenotype. The gene for the porin was found in the multidrug-resistant DR1 (CHQ89_07730), DR2 (CHQ90_00810), and AB067 (A8A08_11745), and the ST195 homologs exhibited only 79% identity with the sequence (A1S_2538) found in *A. baumannii* ATCC 178978 (Figure 2A and Supplementary Figure 3A).

Omp 33-36 (Omp34) is another well conserved and highly immunogenic porin of *A. baumannii* ascribed to induce caspase-dependent apoptosis, enhance bacterial persistence, and cause systemic virulence in infected humans. Although the homologs of Omp33-36 was found in the contigs of DR1 (accession: NOXP01000009.1; CHQ89_12430), DR2 (accession: NOXO01000013.1; CHQ90_11410), and AB067 (accession: LZOC01000011.1; A8A08_14050), with >99% identity to A1S_3297 of *A. baumannii* ATCC 178978, it is of importance to state that the porin from ST49/ST128 had a distinct extended loop in its homology modeled structure, quite different from its counterpart in ST2/ST1816 strains (Figures 2B,C and Supplementary Figure 3B).

The OmpW porin, referred to be highly immunogenic than Omp38, with a role in iron homeostasis, was also detected in these clinical strains DR1 (CHQ89_05870), DR2 (CHQ90_01520), and AB067 (A8A08_09575), displaying 97% identity to A1S_0292 from *A. baumannii* ATCC 17978.

The SurA1, surface antigen porin primarily detected in *A. baumannii* strain CCGGD201101 isolated from chicken, was found present in these MDR strains (DR2: CHQ90_05830 and AB067: A8A08_07245) with 100% identity to KT023081.2.

#### Phospholipases

The virulent factor phospholipase, regulated by ferric uptake transcriptional factor, is phosphodiesterases that break phospholipids of cells either before (phospholipase C) or after (phospholipase D) the phosphate group, which imparts a major role in cellular invasion, serum resistance, the release of inositol triphosphate, overall influencing the *in vivo* pathogenesis. These phospholipases with the KHD motif were found conserved in these MDR strains DR1 (CHQ89_17105, CHQ89_10180), DR2 (CHQ90_16425, CHQ90_12960), and AB067 (A8A08_07850, A8A08_03930) with >99% identity with other members of *A. baumannii*.

#### Nutrient Acquisition Systems

Bacteria exploit the high-affinity (ZnuABC transporters) and low-affinity (cytoplasmic membrane protein with eight transmembranes ZupT) importers and exporters, namely P-type ATPase ZntA and ZitB to scavenge for zinc from the host environment, as it is the required cofactor for DNA repair, replication, protein synthesis, and enzymatic reactions. Sequence analysis revealed the presence of the high-affinity ABC-type transporter (100% identity) in *A. baumannii* strains DR1 (CHQ89_16635, CHQ89_16650, CHQ89_16645), DR2 (CHQ90_08475, CHQ90_08460, CHQ90_08465), and AB067 (A8A08_02190, A8A08_02205, A8A08_02200).

Although copper is required by bacteria for cuproenzyme activity, it uses the *copABCRS* efflux pump as the stress responsive system to combat its toxicity. This entire operon was found encoded only in *A. baumannii* strain DR1 (CHQ89_15120, CHQ89_15100, CHQ89_15095, CHQ89_15115, and CHQ89_15110) with CopB consisting of two MNNM binding motifs. In contrast, the multi-oxidase CopA found in all the strains (CHQ89_12130, CHQ90_15750, and A8A08_05065) had the conserved metal-binding motif HCHLLYHM known to interact with the metal.

Like other metals, iron is also utilized by *A. baumannii* for electron transport chain, tricarboxylic acid cycle, deoxynucleotide biosynthesis, and other biological processes. This bacillus produces high-affinity iron chelators to scavenge iron from the host environment during limited conditions, and the predominant siderophore is the catechol hydroxamate-based *fur*-regulated acinetobactin. The entire operon involved in acinetobactin biosynthesis (*bas*ABCDEFGHIJ), acinetobactin utilization (*bau*ABCDEF), and acinetobactin release (*bar*AB) were detected in the clinical strains DR1 (NODE_5_length: NOXP01000005.1), DR2 (NODE_18_length: NOXO01000018.1), and AB067 (scaffold 15: LZOC01000006.1), respectively, exhibiting > 96% identity with its homologs from *A. baumannii* ATCC 17978.

#### Lipopolysaccharide and Capsule

The structure of surface carbohydrates determines the virulence capability of *A. baumannii*. Capsule forms a discrete layer outside the cell membrane and helps in colonization, persistence, and evading the immune attack and is the well-characterized major virulence factor in this human pathogen. The external capsule production and its export machinery are tyrosine kinase pathway dependent with required proteins clustered in the K-locus of the genome with two conserved and one middle variable region. In contrast, the clinical strain *A. baumannii* DR2 (NODE_9_length_162686_cov_34.9_ID_15) and *A. baumannii* strain AB067 (scaffold8xsize162476) possess the KL3 capsular types with outer membrane protein (*wza*), phosphatase (*wzb*), tyrosine kinase (*wzc*), translocase (*wzx*), and polymerase (*wzy*), exhibiting > 95% identity with 25.4-kb cluster in *A. baumannii* strain ATCC 17978 (CP012004.1), which uses D-GalpNAc (*N*-acetyl-D-galactosamine) as its first sugar.

The gene cluster found in the *A. baumannii* strain DR1 was of the K11 type exhibiting > 95% identity with the region from *A. baumannii* isolate, J9 (KF002790), comprising of four *gtr*26-29 genes (glycosyltransferase), an *itrA3* (initiating transferase), and *atr6* (acetyltransferase) together with the genes encoding the units for polymerization and its transport across the membrane.

The lipopolysaccharide biosynthesis of *Acinetobacter* begins with the sugar moiety, where UDP-GlcNAc is acylated by LpxA, decylated by zinc-dependent LpxC, subsequently resulting in UDP-2,3-diacylglucosamine by LpxD. LpxH generates lipid X (2,3-diacylglucosamine-1-phosphate), which is added to UDP-2,3-diacylglucosamine by LpxB. Subsequently, after a series of enzymatic reactions, Kdo_2_-lipid A is formed, and finally, mature lipopolysaccharides form the outermost layer of the cell. The transferase *lpxA* [UDP-acetylglucosamine acyltransferase], synthase *lpxB* [lipid A-disaccharide synthase], transferases *lpxD* [UDP-3-*O*-[3-hydroxymyristoyl] glucosamine *N*-acyltransferase], and *lpxL* [Kdo2-lipid IVA lauroyltransferase/acyltransferase], lipid A phosphoethanolamine transferase (A1S_2752; *eptA*) were all found in these MDR strains DR1 (OCL8-type), DR2 (OCL1d-type), and AB067 (OCL1d-type) with 100% identity to proteins (A1S_1965, A1S_1668, and A1S_1967) found in *A. baumannii* strain ATCC 17978.

The mutations behind colistin resistance were identified in *lpxA* (Y131H), *lpxB* (C120R; N287O), and *lpxD* (E117K), isochorismate synthase (A96S, D198G, K245R, and I387V), respectively.

The deacetylase *lpxC* [UDP-3-*O*-acyl-*N*-acetylglucosamine deacetylase] found in these strains exhibited 99% identity with A1S_3330, as mutations at position C120R and N287D were also detected.

Additionally, the UDP-23-diacylglucosamine hydrolase (*lpxH*) found in these strains exhibited 95% identity to A1S_2110, as prominent sense mutations were found in position N163D, H217Q, Q219Y, H228N, and L229T.

#### Secretion Systems and Biofilm Formation

Operons encoding the type IV fimbrial assembly protein *pilC* and ATPase *pilB*, along with fimbrial biogenesis protein *pilA*, *fimT*, *pilV*, *pilW*, *pilX*, *pilY*1, *pilM*, *pilN*, *pilO*, *pilP*, and *pilQ*, were detected in the genomes of all the MDR strains, whereas the T6SS gene clusters were found in the genomes of strains DR2 (NODE_11_length_140895) and AB067 (scaffold22| size66907) only.

Previous studies have shown that pilus formation by CsuA/BABCDE usher-chaperone assembly system is pivotal for adherence to human cells and attachment to innate surfaces (Geisinger et al., 2019). A histidine kinase regulatory system bfmRS regulate the entire operon, and all the genes, namely *csuA* and *csuB* that encode for the minor pilin subunits, *csuC* for chaperone, *csuD* for usher, and *csuE* for adhesion, were detected in NODE_2_length (NOXP01000002.1), NODE_5_length (NOXO01000005.1), and scaffold 9 (LZOC01000060.1) of *A. baumannii* strains DR1, DR2, and AB067 respectively, exhibiting > 95% identity with its counterparts from *A. baumannii* ATCC 17978.

The surface protein, BAP, has been associated with biofilm formation and infection process, and this 217-kDa biofilm-associated type I secretion protein was found in NODE_1_length and scaffold 9 of *A. baumannii* strains DR2 and AB067, respectively, which displayed 97.5% identity with its homologs present in other *A. baumannii* strains.

Among the polysaccharides found in *A. baumannii* biofilm is poly-β-(1-6)-*N*-acetylglucosamine, considered an important factor that protects bacteria against host immune response and biofilm formation, and the entire locus *pgaABCD* was found in NODE_2_length (NOXP01000002) NODE_15_length (NOXO01000015) and scaffold 12 (LZOC01000003) of *A. baumannii* strains DR1, DR2, and AB067, respectively, which displayed > 98.64% identity with its homologs present in other *A. baumannii* strains.

The penicillin-binding protein demonstrated to have a role in serum resistance was encoded on the locus (NODE_12_length: NOXP01000012.1, NODE_22_length: NOXO01000022.1, and scaffold23: LZOC01000015.1) in the genomes of strains DR1, DR2, and AB067, respectively, which exhibited > 98.6% identity with homologs from *A. baumannii* ATCC 17978.

### Antibiotic Resistance Genes Present in *Acinetobacter baumannii* Strains

#### Antibiotic Resistome of *Acinetobacter baumannii* Strains DR1, DR2, and AB067

The class A β-lactamase *bla*_*TEM*_*_–_*_1D_ was encoded on locus NODE_55_length: NOXO01000054.1 (CHQ90_18825), in the genome of strain DR2, which exhibited 100% identity with homologs from other *A. baumannii* strains.

The class-C β-lactamase *bla*_*ADC*_ homolog was detected on locus NOXP01000005.1: CHQ89_08490, NOXO01000018.1: CHQ90_14045, and LZOC01000006.1: A8A08_17150 in the genomes of *A. baumannii* strains DR1, DR2, and AB067, respectively, which exhibited 100% identity with homologs from other *A. baumannii* strains.

The class-D β-lactamase of OXA-51 family, *bla*_*oxa–98*_ gene, was detected on locus NOXP01000001.1: CHQ89_00585 in *A. baumannii* strains DR1, and *bla*_*oxa–66*_ gene was identified in NOXO01000019.1: CHQ90_14165; LZOC01000007.1: A8A08_03495 in the genomes of *A. baumannii* strains DR2 and AB067, respectively, which exhibited 100% identity with its respective homologs from other gene *A. baumannii* strains.

The dominant class-D β-lactamase (of the OXA-23 family) in MDR strains was detected on the locus (NOXO01000051.1; CHQ90_18780 and LZOC01000034.1: A8A08_18555) only in the genomes of strains DR2 and AB067, respectively, which exhibited 100% identity with homologs from other *A. baumannii* strains.

The aminoglycoside nucleotidyltransferase ANT(3′′)-IIc gene that confers resistance to streptomycin, spectinomycin, was detected in *A. baumannii* strains DR1 CHQ89_16705, DR2 CHQ90_08400, and AB067 A8A08_02265 respectively, and these genes were found in the genomic island region.

The aminoglycoside phosphotransferases that confer resistance to gentamicin (CHQ90_18865, CHQ90_15195, and A8A08_04525) and streptomycin (CHQ90_15190 and A8A08_04530) were also detected in these MDR strains.

The 16S rRNA methyl transferase, *armA*, ABC ribosomal protection protein *msrE*, and macrolide phosphotransferase *mphE* (CHQ90_05300 and A8A08_06415) were detected in these strains under study.

Quinolone resistance operates by intrinsic mechanisms such as active efflux of drugs, leading to minimal drug accumulation inside cells or presence of transferable determinant *qnr* or due to sense variations in *gyrA* (DNA gyrase) or *parC* (topoisomerase IV). Correlation between quinolone resistance and mutations in the active site and additional sites of these key enzymes, *gyrA* and *parC*, has been well studied in *A. baumannii*. The *gyrA* subunit (S81L) and *parC* subunit (S84L, V104I, and D105E) had mutations in their operons leading to quinolone resistance, as susceptibility testing confirmed the phenotype.

The different types of transposons identified in *A. baumannii* strains DR2 and AB067 are enlisted in Table 1.

**TABLE 1:** Transposable element distribution in genomes *of A. baumannii* DR2 and AB067 strains.

Strain/Scaffold	Transposon type/Accession	% Identity	% Coverage	Length	Query start	Query end	Subject start	Subject end	*E* value	Bit score
***A. baumannii* DR2**										
NOXO01000006.14	Tn6292	100	60.9	771	1	771	495	1265	0	1,391
NOXO01000002.12	Tn6166	100	60.8	10,719	171	10,889	6913	17631	0	19,331
NOXO01000005.11	Tn2007| EF059914	99.87	60.57	1,498	1177	2,674	2473	976	0	2,693
NOXO01000005.11	Tn2008| GQ861438	99.37	62.8	1,597	1327	2,923	2531	942	0	2,837
NOXO01000002.12	Tn6205| CP003505	100	63.58	1,756	6873	8,628	2761	1006	0	3,168
NOXO01000003.10	Tn6022-delta-1| JN247441	100	75.65	6,921	31845	38,765	1	6921	0	12,482
***A. baumannii* AB067**										
scaffold37| size87970	Tn2007| EF059914	99.87	60.57	1,498	1049	2,546	2473	976	0	2,693
scaffold36| size116788	Tn6292	100	64.77	820	22199	23,018	1265	446	0	1,480
scaffold17| size844737	Tn6205| CP003505	100	63.58	1,756	59291	61,046	1006	2,761	0	3,168

*Transposable elements were identified using BacAnt (http://bacant.net/) in A. baumannii strains.*

### Different Efflux Pumps Encoded by *Acinetobacter baumannii* Strains

#### Resistance Nodulation Division Family Efflux Pump

The tripartite system AdeABC is the first characterized RND multidrug efflux pump in *A. baumannii* BM4454 for conferring resistance to aminoglycosides, cefotaxime, quinolones, and tigecycline, whose expression is tightly regulated by *adeRS* operon (Geisinger et al., 2019). The entire *adeABC* locus was found on NOXP01000001, NOXO01000021, and LZOC01000017 in the genomes of strains DR1, DR2, and AB067, respectively, and they exhibited > 92% identity with its homolog from *A. baumannii* strain ATCC 17978.

The *adeB* gene (CHQ89_01920, CHQ90_14855, and A8A08_10810) detected in these strains also harbored mutations in positions G306V, N427S, A562T, A643D, and T646S, respectively, with >92% identity to A1S_1750. On homology modeling comparison, the AdeB protein from ST128 revealed crucial differences (Figure 2D and Supplementary Figure 3C).

The multiple sequence alignment of *adeA* with respect to *E. coli* (based on PDB 5o66) revealed the presence of specific mutations at conserved residue spots in box-1 (G/A), box-3 (TSDG/NSHG), box-4 (QT/PE, S/D), box-5 (F/Y), and box-9 (G/N) (Supplementary Figure 3D).

The cognate regulatory system *adeR* (CHQ90_14865 and A8A08_10820) and *adeS* (CHQ90_14870 and A8A08_10825) found in these strains displayed > 99% identity compared with ATCC proteins (A1S_1753 and A1S_1754) and also had mutations at position V120I, A136V and L172P, G186V, N268H, Y303F, and V348I, whereas the *adeRS* found in DR1 (CHQ90_14865 and CHQ89_01905) exhibited merely 60% sequence identity, which is interesting.

The *adeIJK* is another efflux pump characterized to have a role in conferring resistance against aztreonam, cephalosporins, fluoroquinolones, tetracycline, and fusidic acid (Geisinger et al., 2019). The *adeIJK* locus was found in the genomes of strains DR1 (NOXP01000007.1), DR2 (NOXO01000017.1), and AB067 (LZOC01000005.1), which exhibited > 99% identity with its homologs from *A. baumannii* ATCC 17978.

The *adeFGH* locus that demonstrated to exhibit resistance to clindamycin, chloramphenicol, fluoroquinolones, and trimethoprim is present in the genomes of *A. baumannii* strains DR1 (NOXP01000002.1), DR2 (NOXO01000005.1), and AB067 (LZOC01000060.1), respectively, with > 97% identity with homologs from *A. baumannii* ATCC 17978. The adjacent transcription regulator protein of this MDR efflux pump cluster *adeL* was detected in these MDR strains (CHQ89_03455, CHQ90_04740, and A8A08_14695) with 100% identity to A1S_2303 from *A. baumannii* ATCC 17978.

The *abeD* efflux pump with a role in osmotic and oxidative stress tolerance besides AMR was detected in these strains (CHQ89_17860, CHQ90_01250, and A8A08_11305), with 100% sequence identity with A1S_2660.

#### Small Multidrug Resistance Family Efflux Pump

The small multidrug resistance (SMR) type efflux pump *abeS* was primarily characterized in *A. baumannii* strain AC0037 and found to be involved in conferring resistance to chloramphenicol, ciprofloxacin, erythromycin, and novobiocin and dyes, and antiseptics (Geisinger et al., 2019). The *abeS* locus was detected in these MDR strains DR1 (NODE_2_length: NOXP01000002.1), DR2 (NODE_5_length: NOXO01000005.1), and AB067 (scaffold 9: LZOC01000060.1), which exhibited > 99% identity with homologs from *A. baumannii* ATCC 17978. The gene has mutations in the 3rd TMH region containing residues 55–77 (M55I) and 4th TMH region, 88–105 (V84L, T91A).

#### Major Facilitator Superfamily Family Efflux Pump

The major facilitator superfamily (MFS) efflux pump *amvA* shown to display decreased susceptibility for ciprofloxacin, erythromycin, norfloxacin, novobiocin, and hospital-based disinfectants such as benzalkonium chloride (Geisinger et al., 2019) was detected in these MDR *A. baumannii* strains DR1 (NODE_10_length: NOXP01000010.1), DR2 (NODE_26_length: NOXO01000026.1), and AB067 (scaffold 28: LZOC01000020.1) with mutations in positions P35A, M245T, and S432N, and one mutation in TMH region at L471I, displaying > 96% identity with homologs from other *A. baumannii* strains.

The transcriptional regulator of the AcrR family, situated upstream of this efflux pump in strains DR1 (CHQ89_14010), DR2 (CHQ90_16410), and AB067 (A8A08_07835), exhibited > 99% identity to A1S_2058 from *A. baumannii* ATCC 17978.

The acquired efflux system *craA* and *tetA* with 12 TMH and *cmlA*, *qacE* were also detected in these *A. baumannii* strains.

#### Multidrug and Toxic Compound Extrusion Family Efflux Pump

The multidrug and toxic compound extrusion (MATE) family efflux pump AbeM, with 448 amino acids, was previously demonstrated to have a role in mediating resistance to norfloxacin, ofloxacin, ciprofloxacin acriflavine, triclosan, doxorubicin, and ethidium bromide (Geisinger et al., 2019). The *abeM* locus found encoded on locus (NODE_3_length: NOXP01000003.1, NODE_2_length: NOXO01000002.1, and scaffold 7: LZOC01000058.1) in strains DR1 DR2 and AB067 exhibited > 99% identity with homologs from other *A. baumannii* strains.

#### Adenosine Triphosphate Binding Cassette Superfamily Family Efflux Pump

The adenosine triphosphate (ATP) binding cassette superfamily (ABC) transporters, also known as ATP binding cassette transporters, exist in all forms of life, their primary function being export/import (importers/exporters) of substrates. Analysis of the available crystal structures classifies these transporters into seven families (type I to VII) with ideal folds distinct from each other. The atypical non-canonical MacB efflux transporter belongs to the exporter class and type VII super family (subfamily 3.A.1.122). The MacB monomer consists of four transmembrane helices and the nucleotide-binding domain and large periplasmic domain. The periplasmic domain has three sub domains—one Saber region, which is alpha/beta rich, and two Porter regions made up of two β-α-β motifs. Efforts made by researchers on the structural data have referred to the mechanism of efflux as “mechanotransmission” or “vacuum cleaner like function.” A previous study (Geisinger et al., 2019) suggests that *A*. *baumannii* exhibited around 4.4-fold increase in gene expression of *macB* in the presence of 100 μg/ml tannic acid, and this transporter was found to be conserved, except a mutation at V488I, in *A. baumannii* strains DR1 (CHQ89_04745), DR2 (CHQ90_11845), and AB067 (A8A08_00665).

#### Signal Transduction System

The different two-component signal transduction proteins detected in these strains are shown in Supplementary Figure 4 along with their domains.

PmrA/PmrB is the well-studied TCS in *A. baumannii* linked to colistin resistance (Geisinger et al., 2019). An independent study revealed that the upregulation of *pmrA* and *pmrB* is a prime mechanism involved in colistin resistance among *A. baumannii* isolates. The *pmrAB* locus was identified on (NOXP01000007.1, NOXO01000017.1, and LZOC01000005.1) in the genomes of strains DR1, DR2, and AB067, respectively, which exhibited > 99% identity with homologs from *A. baumannii* ATCC 17978. The *pmrB* has mutations at A138T, I235T, and A444V, whereas *pmrA* was found conserved.

The biofilm formation is a quintessential feature for the survival of the nosocomial pathogen *A. baumannii* in hostile and desiccated environments. BfmR, which plays the role of RR in TCS BfmRS, is an important factor in regulating cell morphology and biofilm-forming capability of *A. baumannii* 19606 cells. The *bfmRS* locus was found on NOXP01000008.1, NOXO01000012.1, and LZOC01000001.1 in strains DR1, DR2, and AB067, respectively, which exhibited > 99% identity with homologs from *A. baumannii* ATCC 17978.

GacSA has recently been studied in *A. baumannii* to regulate various virulence factors, including motility, pili synthesis, biofilm formation, and serum proteins. The *gacAS* locus was detected on NOXP01000012.1, NOXO01000022.1, and LZOC01000015.1 in the genomes of strains DR1, DR2, and AB067, respectively, with >98% identity with a homolog from *A. baumannii* ATCC 17978 and mutations at I65V, R170G, D389V, and T438P, respectively.

The role of BaeSR in response to exposure to various chemical substances was also studied, and it was noticed that *A. baumannii* was vulnerable to a number of chemical compounds especially tannic acid, after the *baeR* deletion in its system. BaeSR in *A. baumannii* has been considerably shown to dispose of chemicals such as tigecycline and tannic acid by regulating efflux pumps without evidence of direct DNA binding. The *baeRS* locus was encoded (NOXP01000007.1, NOXO01000016.1, and LZOC01000004.1) in the genomes of strains DR1, DR2, and AB067, respectively, with a mutation at S437T and >98.61% identity with a homolog from *A. baumannii* ATCC 17978.

Additional mutations in signaling proteins were also detected, e.g., quorum sensing system *qseB*: T58A, S122N, *qseC* at E128Q, E327G, *phoB* at Q430L, and *gntR* regulator at C113S, N117D, H127D, and S192T in these MDR strains.

The presence of bacterial tyrosine kinase in *A. baumannii* strain AB067 was partly discussed in our previous report (Srinivasan and Rajamohan, 2020b).

### Phenotypic Characterization of *Acinetobacter baumannii* Strains DR1, DR2, and AB067

#### Antibiotic Susceptibility Testing and Efflux Assays

**FIGURE 3:**
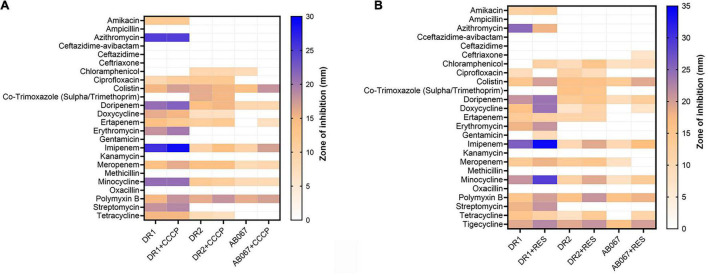
Heat map represents antibiotic resistance of *A. baumannii* DR1, DR2, and AB067 strains. Antibiotic susceptibility testing was performed with different antibiotics in presence and absence of efflux pump inhibitor, **(A)** carbonyl cyanide m-chlorophenyl hydrazone (CCCP), and **(B)** reserpine against *A. baumannii* DR1, DR2, and AB067 strains. Data were tabulated and analyzed, and a heat map was constructed in GraphPad Prism software using mean zone of inhibition values of Kirby–Bauer assays to compare resistance and inhibition pattern among strains.

As per the disk diffusion assay, the *A. baumannii* strains DR1, DR2, and AB067 were found resistant to antibiotics ceftazidime-avibactam, ceftazidime, ceftriaxone, chloramphenicol, co-trimoxazole, and oxacillin, and ST195 variants were additionally resistant to amikacin, ampicillin, azithromycin, kanamycin, methicillin, erythromycin, gentamicin, streptomycin, and tetracycline (Figure 3A).

The MIC values determined using E-strips indicated that strains were highly resistant to different classes of antibiotics, chloramphenicol, erythromycin, fosfomycin, minocycline, nitrofurantoin, oxacillin, and tetracycline, and the ST195 strains were highly resistant to azithromycin, ciprofloxacin, doripenem, ertapenem, faropenem, and gentamicin (Table 2).

**TABLE 2:** Determination of minimum inhibitory concentration of antimicrobial agents in *A. baumannii* strains.

Antibiotics	DR1	DR2	AB067
	
	MIC (μg/ml)		
Amikacin	6	>256	>256
Azithromycin	1	>256	>256
Bacitracin	128	>256	>256
Chloramphenicol	96	>256	>256
Ciprofloxacin	0.25	>256	>256
Colistin	1	1	1
Doripenem	0.5	>32	>32
Doxycycline	0.75	128	>256
Ertapenem	1.5	>32	>32
Erythromycin	16	>256	>256
Faropenem	4	>32	>32
Fosfomycin	>1,024	>1,024	>1,024
Gentamicin	0.5	>1,024	>1,024
Minocycline	25	> 256	>256
Nitrofurantoin	>512	>512	>512
Oxacillin	>256	>256	>256
Tetracycline	16	>256	>256
2,6 DNP	64	256	256
Acridine orange	4	256	256
Acriflavine	0.5	64	64
Ethidium bromide	<0.5	256	256
Rhodamine	<0.5	4	4
Saffranine	<0.5	256	256
SDS	16	>512	>512
Benzalkonium chloride	<0.5	4	4
Chlorhexidine	<0.5	0.5	0.5
Triclosan	<0.5	<0.5	<0.5

*MIC concentrations were deduced using HiMedia E-strips for different antibiotics such as amikacin, azithromycin, bacitracin, chloramphenicol, ciprofloxacin, colistin, doripenem, doxycycline, ertapenem, erythromycin, faropenem, fosfomycin, gentamicin, minocycline, nitrofurantoin, oxacillin, and tetracycline, for structurally unrelated compounds such as acridine orange, acriflavine, ethidium bromide, rhodamine, saffranine, and SDS, and hospital-based disinfectants such as benzalkonium chloride, chlorhexidine, and triclosan; MIC values were tested using agar dilution method.*

The involvement of active efflux was evaluated in the presence and absence of efflux pump inhibitors, and in the presence of reserpine, the strain exhibited sensitivity toward antibiotics, mainly doripenem, doxycycline, imipenem, meropenem, minocycline, and tetracycline (Figure 3B).

**FIGURE 4:**
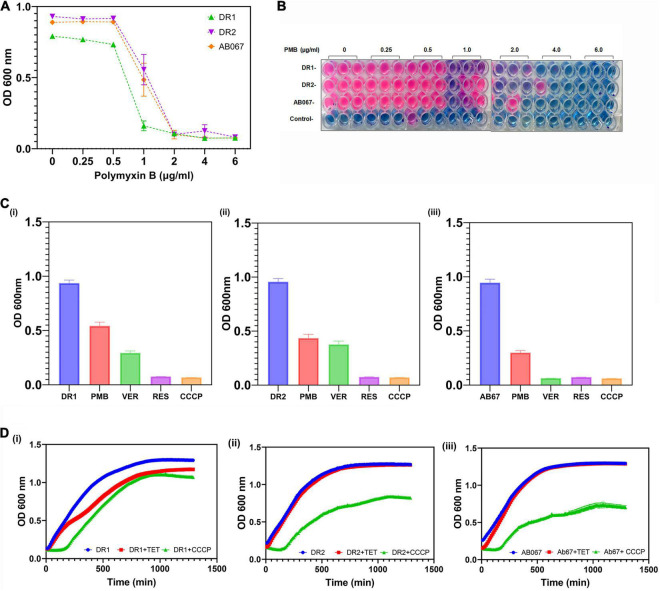
Determination of MIC for polymyxin and growth inactivation assays. **(A)** Growth of *A. baumannii* DR1, DR2, and AB067 strains was evaluated in presence of different polymyxin concentrations. Optical density of growth was plotted against a range of polymyxin concentrations to determine growth and minimum inhibitory concentrations of polymyxin. **(B)** Resazurin microtiter assay was performed to evaluate cell viability of *A. baumannii* DR1, DR2, and AB067 strains in presence of various concentrations of polymyxin (PMB). After period of incubation for 15 h at 37°C, PrestoBlue, a resazurin-based cell viability reagent (Invitrogen), was added to wells containing *A. baumannii* DR1, DR2, and AB067 strains and control (only media) and further incubated as per manufacturer’s protocol. After period of incubation, color change from natural resazurin blue to pink (reduced form) in wells was scored to determine MIC; no color change indicates no bacterial growth. **(C)** Growth inhibition assay was performed in presence and absence of efflux inhibitors verapamil (VER), CCCP, and reserpine (RES) (each of 5 μg/ml) with polymyxin in DR1 **(i)**, DR2 **(ii)**, and AB067 **(iii)** strains. **(D)** Growth inhibition assay was performed in presence of tetracycline as substrate for *A. baumannii* strains DR1 **(i)**, DR2 **(ii)**, and AB067**(iii)**, tabulated and analyzed using GraphPad Prism software.

The MIC for polymyxin was determined in LB broth, and the role of active efflux in mediating polymyxin resistance was also determined, as shown in Figures 4A–C.

The possible involvement of active efflux in conferring tetracycline resistance was confirmed in the presence of different concentrations of CCCP in these resistant strains *A. baumannii* (Figure 4D). The growth inhibition assay was performed in the presence of reserpine, phenylalanine-arginine beta-naphthylamide, conclusively indicating the role of efflux pumps in mediating tetracycline resistance, in these clinical strains (data not shown).

The strains were tested for biofilm formation using crystal violet staining, and it was observed that ST195 strains had the ability to form biofilm on polystyrene tubes.

The strains that were also tolerant to hospital-based disinfectants, such as benzalkonium chloride (up to 4 μg/ml), triclosan (0.5 μg/ml), and chlorhexidine (0.5 μg/ml), exhibited reduced growth by 3.29-fold at 9 h when grown in the presence of pump substrate EtBr and inhibitor CCCP (5 μg/ml) (data not shown), overall, suggesting that active efflux remains the predominant mechanism for AMR in these CRAB Indian clinical strains.

#### Phenotype Microarray Analysis of *Acinetobacter baumannii* Strains

**FIGURE 5:**
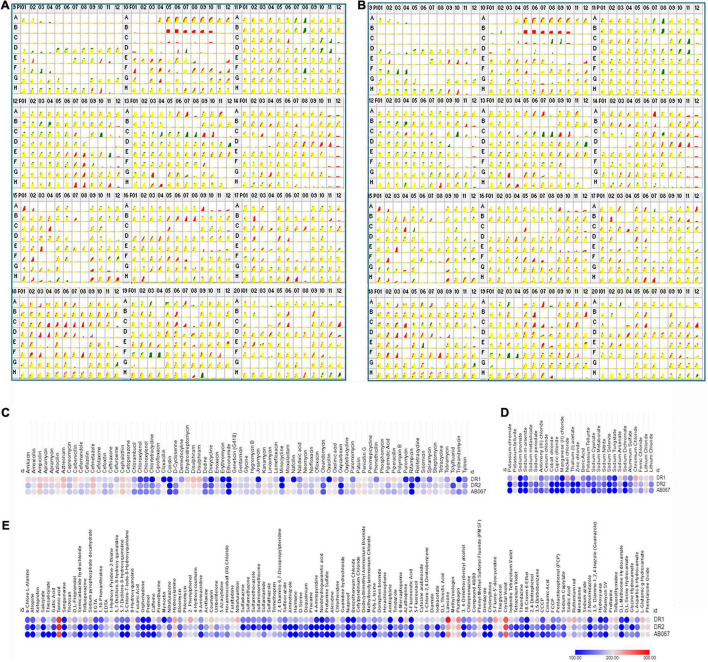
Comparative profiles of Biolog Phenotype MicroArray analysis of *A. baumannii* DR1, DR2, and AB067 strains. Phenotypic microarray analysis of *A. baumannii* DR1, DR2, and AB067 strains to determine metabolic utilization profiles using PM 09 to PM20 plates. Kinetic growth response curve for *A. baumannii* DR1 and DR2 **(A)** and *A. baumannii* DR1 and AB067 **(B)** strains in PM09 to PM20 plates (top panel left as PM09 to right bottom end panel as PM20). Growth kinetics of *A. baumannii* DR2 shown in green, compared with DR1 strain in red. Yellow indicates equivalent growth response or overlapping of DR1 and DR2 kinetic curves. Red kinetic response curves indicate a stronger response by DR1 strain, whereas green indicates a stronger growth response by DR2 strain. Heat maps of selected phenotype microarray profiles of *A. baumannii* DR1, DR2, and AB067 strains for antimicrobial compounds **(C)**, toxic **(D)**, and miscellaneous compounds **(E)**. Each dot represents Omnilog values representing all time points of bacterial growth. Omnilog values of 100 or more were considered for phenotype analysis. Gradient heat maps were generated among strains for each condition using Omnilog values in Morpheus online tool (https://software.broadinstitute.org/morpheus), which displayed blue color for relative lower growth, and red color represents high growth phenotype.

The Biolog PM assays are based on a bacterial respiration system determined by utilizing tetrazolium redox dye that can simultaneously test a large number of phenotypic traits (Bochner, 2009). The phenome of *A. baumannii* DR1, DR2, and AB067 were investigated with the Biolog PM system using PM09–PM20, 96 well plates comprising of 1,152 compounds to test the tolerance of osmolytes (PM09), pH conditions (PM10), antibiotics, and chemical sensitivity (PM11–PM20). The metabolically active conditions of strains that grow on different substrates were scored and analyzed (Figures 5A,B).

The *A. baumannii* strains were examined for osmo and pH tolerance. The strains exhibited sensitivity growth at pH 4.5 and lower; however, these strains were able to deaminate a few compounds at pH 4.5. The DR1 strain exhibited higher respiration activity at pH 4.5 compared with *A. baumannii* DR2 and *A. baumannii* AB067. Moreover, all the strains exhibited good growth at pH 4.5 in the presence of an acidic pH growth enhancer L-norvaline, which signifies that the *A. baumannii* strains have the ability to grow at acidic pH. In addition, these strains have the ability to grow at alkaline pH (pH 5–10), indicating that strains may be evolved with survival strategies to grow in extreme alkaline conditions.

Furthermore, these strains displayed growth at 1–4% of sodium lactate, 20–200 mM of sodium phosphate, 40–100 mM of sodium nitrate, and 10–60 mM of sodium nitrite.

The strains were examined for antibiotics and chemical sensitivity against 960 compounds using PM plates (PM11–PM20). The *A. baumannii* strain DR1 was sensitive to a wide range of antimicrobial agents. Furthermore, it exhibited gained phenotype for folate synthesis, PABA analog, folate pathway antagonists, and pH decarboxylase substrates.

On comparing *A. baumannii* DR1, the *A. baumannii* strains DR2 and AB067 strains displayed gained phenotypic resistance to compounds such as polymyxin B, doxycycline, chlortetracycline, minocycline, rolitetracycline, cloxacillin, nafcillin, oxacillin, phenethicillin, erythromycin, and D,L-serine hydroxamate (Figure 5C).

Conversely, where the *A. baumannii* strains DR2 and AB067 exhibited sensitivity toward atropine, salicylate, semicarbazide hydrochloride, 5,7-dichloro-8-hydroxyquinoline, 5-chloro-7-Iodo-8-hydroxyquinoline, caffeine, umbelliferone, sulfadiazine, sulfathiazole, sulfamethoxazole, sulfanilamide, sulfisoxazole, harmane, dequalinium, procaine, alexidine, dodecyltrimethyl ammonium bromide, poly-L-lysine, tinidazole, 3, 4-dimethoxybenzyl alcohol, ornidazole, rifampicin, potassium chromate, sodium *m*-arsenite, cobalt chloride, cupric chloride, zinc chloride, sodium dichromate, and glycine hydroxamate, it is interesting to state that *A. baumannii* DR1 strain exhibited gained resistance phenotype toward these compound and metals compounds (Figures 5C–E).

However, no growth was observed in all three strains for compounds such as novobiocin, 5-fluoroorotic acid, 2,2’-dipyridyl, potassium chromate, cadmium chloride, sodium metavanadate, sodium orthovanadate, fusidic acid, dichlofluanid, protamine sulfate, rifamycin SV, lidocaine, and captan (Supplementary Figures 5A–C).

The observed phenotypic patterns suggest that the *A. baumannii* strains DR2 and AB067 were able to grow in the presence of a range of antimicrobial compounds that indicate the ability of these strains to adapt itself in diverse conditions, and dissemination of such highly tolerant strains in hospital settings may limit the available antimicrobial therapeutic options. Additionally, *A. baumannii* DR1 strain displayed a higher respiration rate on folate synthesis, pH, decarboxylase substrates, and toxic compounds, overall indicating its ability to sustain in selective pressure conditions, importantly toxic metal compounds that are known risk factors to inducing AMR, therefore monitoring the changing phenotypic behavior of such novel ST variants (ST128) becomes significant.

#### Relative Expression of Antimicrobial Resistance Determinants

**FIGURE 6:**
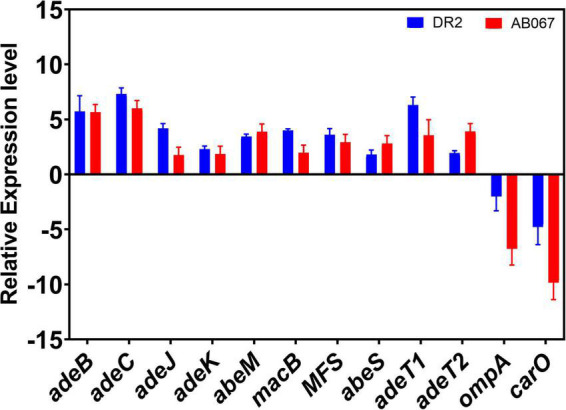
Relative expression levels of antibiotic-resistant determinants; efflux pumps, outer membrane protein, and porins in CRAB strains. *A. baumannii* DR1 strain was used as a reference control.

In this study, we examined the relative expression differences in carbapenem-resistant isolates *A. baumannii* DR2 and *A. baumannii* AB067 compared with *A. baumannii* DR1. The relative gene expression of antibiotic-resistant determinants such as efflux pumps, porins, and outer membrane protein in *A. baumannii* DR2 and AB027 strains is shown in Figure 6. The expression of efflux pumps was significantly upregulated two- to sevenfold in DR2 and AB067 as compared with the DR1 strain. Among the efflux genes, the expression of *adeB* and *adeC* were highest with five- to sevenfold in DR2 and AB067 strains compared with DR1, a carbapenem susceptible isolate. However, *adeJ* and *adeK* expression were found to be increased by two- to fourfold, respectively, in the DR2 strain, whereas twofold was observed in the AB067 strain. We also found the two- to threefold increased expression of *abeM*, *macB*, MFS (*ampG* homolog), and *abeS* in both the strains, and interestingly, the expression of *adeT1* (ABAYE0008 homolog) and *adeT2* (ABAYE0010 homolog) were higher by three- to sixfold in *A. baumannii* DR2 and *A. baumannii* AB067 isolates. The relative expression of *ompA* and *carO* were found to be lowest with three to ninefold (*p* = 0.001) in DR2 and AB067 isolates, and these data corroborate with the modeled structure where mutations partly blocked the porin channel. Different carbapenem susceptible isolates DR1, DR2, and AB067 exhibit differences in gene expression, indicating that the quantifying expression levels of efflux genes and other predominant resistance genes can be one of the useful tools to diagnose and for effective treatment of CRAB isolates in clinical settings.

## Conclusion

Upsurge in AMR phenotype in human pathogens has left physicians with limited therapeutic options. *A. baumannii* is a pleomorphic bacillus with natural competent behavior, responsible for causing nosocomial infections to severe bloodstream infections and pneumonia. The *A. baumannii* resists hospital sterilization procedures and survives on abiotic surfaces for long, and with its ability to acquire diverse resistance islands/determinants, this superbug often displays huge genetic plasticity and heterogeneity. The WHO has ranked CRAB among its “top critical threat” pathogens, and outbreak clones usually belong to the international clonal groups I, II, and III. The occurrence and dissemination of *bla*_*OXA23*_ harboring CRAB, from IC-II (ST2), have increased significantly worldwide in recent years, including in India.

Here, we have reported the genotypic and phenotypic characterization of drug-resistant *A. baumannii* strains from India that belongs to novel sequence variants. This is the first study to report the characterization of ST128 *A. baumannii* for the very first time, with a variant form of key resistance genes, i.e., Omp33-36, CarO, AdeABC, and AdeRS.

The findings of this study not only highlights the alarming coexistence of several resistance determinants, i.e., *bla*_*OXA–23*_, *bla*_*OXA–66*_, *bla*_*ADC73*_, and *bla*_*TEM–*1D_, in one genome, rather emphasize the pressing need to perform regular epidemiological surveillance of clinical *A. baumannii* strains with coordinated networking, paramount for the growing AMR problem, so that novel alarming variants such as ST128 does not remain underrated or enigmatic, which completely holds the potential to cause a possible untreatable outbreak, as reflected by its unique genetic makeup, genome organization, and resistant phenotype.

## Data Availability Statement

The genome sequence data has been deposited in NCBI Genbank accession numbers LZOC01, NOXO01, and NOXP01.

## Author Contributions

SB and GR conceived the idea, designed the strategy, performed the required analysis in this study, and wrote the manuscript. MV performed the drug susceptibility assays. All authors have read and finally approved this report.

## Conflict of Interest

The authors declare that the research was conducted in the absence of any commercial or financial relationships that could be construed as a potential conflict of interest.

## Publisher’s Note

All claims expressed in this article are solely those of the authors and do not necessarily represent those of their affiliated organizations, or those of the publisher, the editors and the reviewers. Any product that may be evaluated in this article, or claim that may be made by its manufacturer, is not guaranteed or endorsed by the publisher.

## Footnotes

1
https://www.pewtrusts.org/en/research-and-analysis/data-visualizations/2014/antibiotics-currently-in-clinical-development

2
http://www.bioinformatics.babraham.ac.uk/projects/fastqc

3
http://pgrc.ipk-gatersleben.de/misa

4
https://realphy.unibas.ch/

5
https://itol.embl.de

6
http://phaster.ca

7
https://card.mcmaster.ca/analyze/rgi

## Supplementary Material

Supplementary Figure 1 Functional annotations of core and accessory genes of *A. baumannii* strains. **(A)** The COG functional categories has been plotted against the COG assigned proteins for *A. baumannii* DR1, DR2, and AB067 strains are shown here. **(B)** The gene annotation data as per RAST program. The proteins were classified under different functional class namely membrane transport (5.13, 3.93, and 3.97%), stress response (3.67, 4.25, and 4.25%), virulence, disease and defense (3.17, 2.83, and 2.79%), regulation and cell signaling (2.09, 2.97, and 3.006%), cell wall and capsule (1.39, 4.46, and 4.58%), and iron acquisition and metabolism (1.26, 1.02, and 1.07%) for *A. baumannii* DR1, DR2, and AB067 respectively.

Supplementary Figure 2 Distribution of prophages in *A. baumannii* strains. **(A)** The DR1 has six prophages of size 34131, 55217, 77242, 126717, 49042, and 30064 bp with GC content ranging from 37.71 to 39.66%. DR1 prophage consists of genes encoding copper resistance protein CopD, copper resistance protein CopC, copper-translocating P-type ATPase, CusA/CzcA family heavy metal efflux RND transporter copper-binding protein, ferrous iron transporter B, ferrous iron transport protein A, MerR family transcriptional regulator and Biofilm associated protein. **(B)** The DR2 has four prophages of size 52055, 87316, 8894, and 70664 bp, with GC content ranging from 36.92 to 41.19% and also a type VI related region of 19412 bp. **(C)** The AB067 has four prophages of size 64203, 84818, 51529, and 65238 bp, with GC content ranging from 36.92 to 40.61% and also a type VI related region of 19412 bp. The DR2 and AB067 clusters consists of genes encoding aminoglycoside *O*-phosphotransferase APH(6)-Id, APH(3”) family aminoglycoside O-phosphotransferase, OXA-23 family carbapenem-hydrolyzing class D beta-lactamase, Msr family ABC-F type ribosomal protection protein, universal stress protein, acyltransferase, catalase, and integrase.

Supplementary Figure 3 Multiple-sequence alignment of resistance determinants from *A. baumannii* strains. Sequence alignments of homologs of CarO [**A**, CHQ89_07730 (DR1), CHQ90_00810 (DR2), A8A08_11745 (AB067)], Omp33–36 [**B**, CHQ89_12430 (DR1), CHQ90_11410 (DR2), A8A08_14050 (AB067)], *adeA* [**C**, NOXP01000001 (DR1), NOXO01000021 (DR2) and LZOC01000017 (AB067)], *adeB* **(D)** were performed using CLUSTAL Omega (https://www.ebi.ac.uk/Tools/msa/clustalo) and formatted in ESPript server (http://espript.ibcp.fr/ESPript/cgi-bin/ESPript.cgi). The secondary structural elements are shown on each of the alignments using PDB templates of 4fuv.1 for CarO **(A)**, 6gie.1 for Omp33-36 **(B)**, 7kge.1 for AdeB **(C)** and *E. coli* AcrA structure (PDB 5o66) for AdeA **(D)**. The mapping of boxes 1–9 in AdeA sequence alignment represents probable interaction boxes as described in McNeil et al. (2019). The arrows indicate a β-sheet, the coils indicate α-helices, TT indicates β turns, and η indicates 310 helices. Residues strictly conserved have a colored background and are indicated by boldface letters; residues conserved between groups are boxed.

Supplementary Figure 4 The TCS domain organization in *A. baumannii.* The different signaling proteins found in *A. baumannii* strains DR1, DR2, and AB067 are depicted in here. The different signaling proteins chosen in *A. baumannii* strains DR1, DR2, and AB067 are homologs of proteins found in *A. baumannii* ATCC 17898; namely A1S_0234 Helix-turn-helix, Fis-type, A1S_0235 sensor protein, A1S_0236 BarA-associated response regulator UvrY, A1S_0260 AlgZ/FimS, A1S_0261 positive alginate biosynthesis regulatory protein, A1S_0574 signal transduction histidine-protein kinase BarA, A1S_0748 transcriptional response regulator, OmpR family, A1S_0749 histidine kinase, A1S_1393, A1S_1394 LuxR family, A1S_1753 LuxR family, A1S_1754 histidine kinase, A1S_1977 nitrogen regulation protein NtrB, A1S_1978 NtrC, A1S_2006 response regulator NasT, A1S_2137 DNA-binding response regulator, A1S_2138 histidine kinase, A1S_2287 Sensor protein *qseC*, A1S_2750 histidine kinase, A1S_2751 LuxR family, A1S_2811 twitching motility protein PilG, A1S_2814 twitching motility protein PilH, A1S_2815 twitching motility protein PilG, A1S_2883 response regulator BaeR, A1S_2884 sensory histidine kinase BaeS, A1S_2906 sensor histidine kinase, A1S_2937 two-component system response regulator QseB, A1S_2938 copper sensory histidine kinase CusS, A1S_3229 response regulator Omp, A1S_3230 osmolarity sensory histidine kinase EnvZ, A1S_3302 sensor histidine kinase, A1S_3304 LuxR family, A1S_3374 regulatory protein PhoB, A1S_3376 phosphate regulon sensor protein PhoR respectively.

Supplementary Figure 5 Phenotype microarray analysis of *A. baumannii* DR1, DR2, and AB067 strains. Phenotypic microarray (PM) analysis of *A. baumannii* DR1, DR2, and AB067 strains were performed to determine the metabolic utilization profiles using PM09 to PM20 plates. Novel Sequence Types in *Acinetobacter baumannii* kinetic growth response curve for *A. baumannii* DR1 and DR2 **(A)** and *A. baumannii* DR1 and AB067 **(B)** strains in PM09 to PM20 plates (top panel left as PM09 to right bottom end panel as PM20). The PM data of PM09 to PM20 plates of DR2 (green color) was compared with DR1 (red color), yellow color indicates the equivalent or overlapping growth response of DR1 and DR2 strains. Few wells were enclosed with boxes indicate that the wells were found to be significantly different. The phenotype microarray data of *A. baumannii* DR1, DR2, and AB067 strains for PM09–PM20 plates were analyzed and depicted in heat map profiles **(C)**. Each dot represents Omnilog values represented all time points of bacterial growth. The Omnilog values were considered for phenotype analysis. The gradient heat maps were generated for *A. baumannii* DR1, DR2, and AB067 strains for each PM plates from PM09 to PM20 using Omnilog values in Morpheus online tool (https://software.broadinstitute.org/morpheus) which displayed blue color for relative lower growth and red color represents high growth phenotype.
