# Supplementary figures and images for: Genotypic and Phenotypic Characterization of Novel Sequence Types of Carbapenem-Resistant Acinetobacter baumannii, With Heterogeneous Resistance Determinants and Targeted Variations in Efflux Operons
Source: Front Microbiol. 2021 Dec 23;12:738371. doi: 10.3389/fmicb.2021.738371 (PMC8735875; doi:10.3389/fmicb.2021.738371)

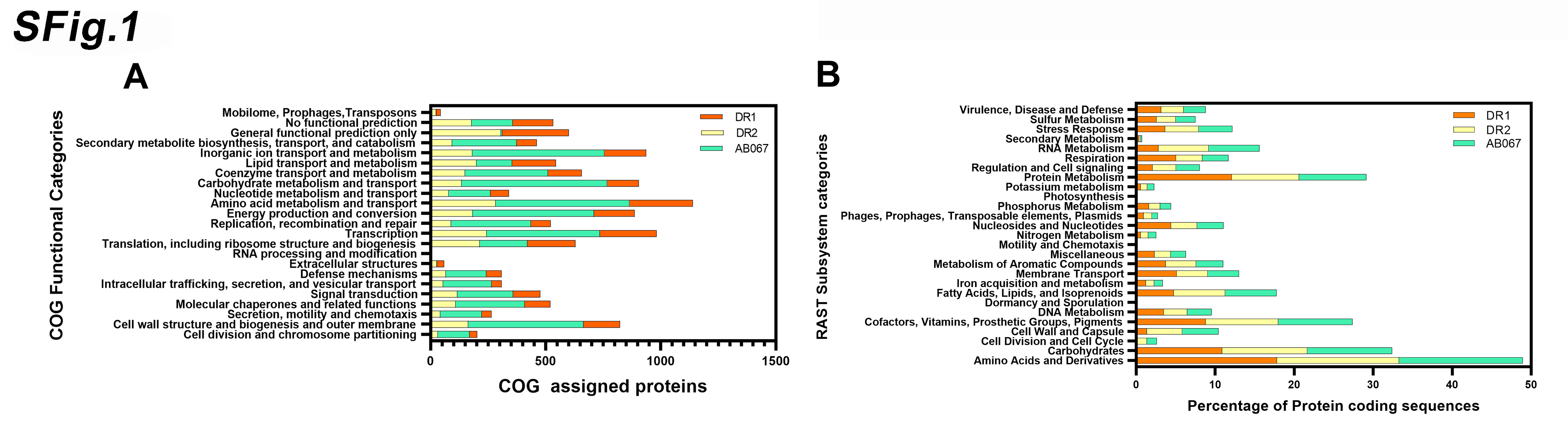

Supplement: Supplementary Figure 1 — Functional annotations of core and accessory genes of A. baumannii strains. (A) The COG functional categories has been plotted against the COG assigned proteins for A. baumannii DR1, DR2, and AB067 strains are shown here. (B) The gene annotation data as per RAST program. The proteins were classified under different functional class namely membrane transport (5.13, 3.93, and 3.97%), stress response (3.67, 4.25, and 4.25%), virulence, disease and defense (3.17, 2.83, and 2.79%), regulation and cell signaling (2.09, 2.97, and 3.006%), cell wall and capsule (1.39, 4.46, and 4.58%), and iron acquisition and metabolism (1.26, 1.02, and 1.07%) for A. baumannii DR1, DR2, and AB067 respectively. [file Image_1.TIF]

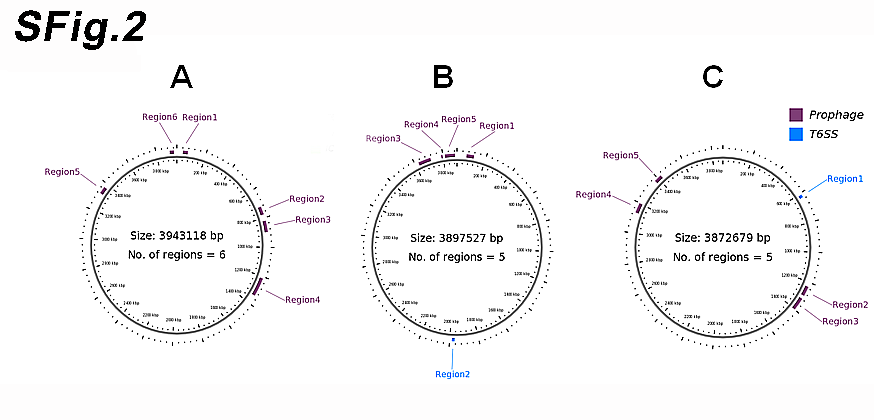

Supplement: Supplementary Figure 2 — Distribution of prophages in A. baumannii strains. (A) The DR1 has six prophages of size 34131, 55217, 77242, 126717, 49042, and 30064 bp with GC content ranging from 37.71 to 39.66%. DR1 prophage consists of genes encoding copper resistance protein CopD, copper resistance protein CopC, copper-translocating P-type ATPase, CusA/CzcA family heavy metal efflux RND transporter copper-binding protein, ferrous iron transporter B, ferrous iron transport protein A, MerR family transcriptional regulator and Biofilm associated protein. (B) The DR2 has four prophages of size 52055, 87316, 8894, and 70664 bp, with GC content ranging from 36.92 to 41.19% and also a type VI related region of 19412 bp. (C) The AB067 has four prophages of size 64203, 84818, 51529, and 65238 bp, with GC content ranging from 36.92 to 40.61% and also a type VI related region of 19412 bp. The DR2 and AB067 clusters consists of genes encoding aminoglycoside O-phosphotransferase APH(6)-Id, APH(3”) family aminoglycoside O-phosphotransferase, OXA-23 family carbapenem-hydrolyzing class D beta-lactamase, Msr family ABC-F type ribosomal protection protein, universal stress protein, acyltransferase, catalase, and integrase. [file Image_2.TIF]

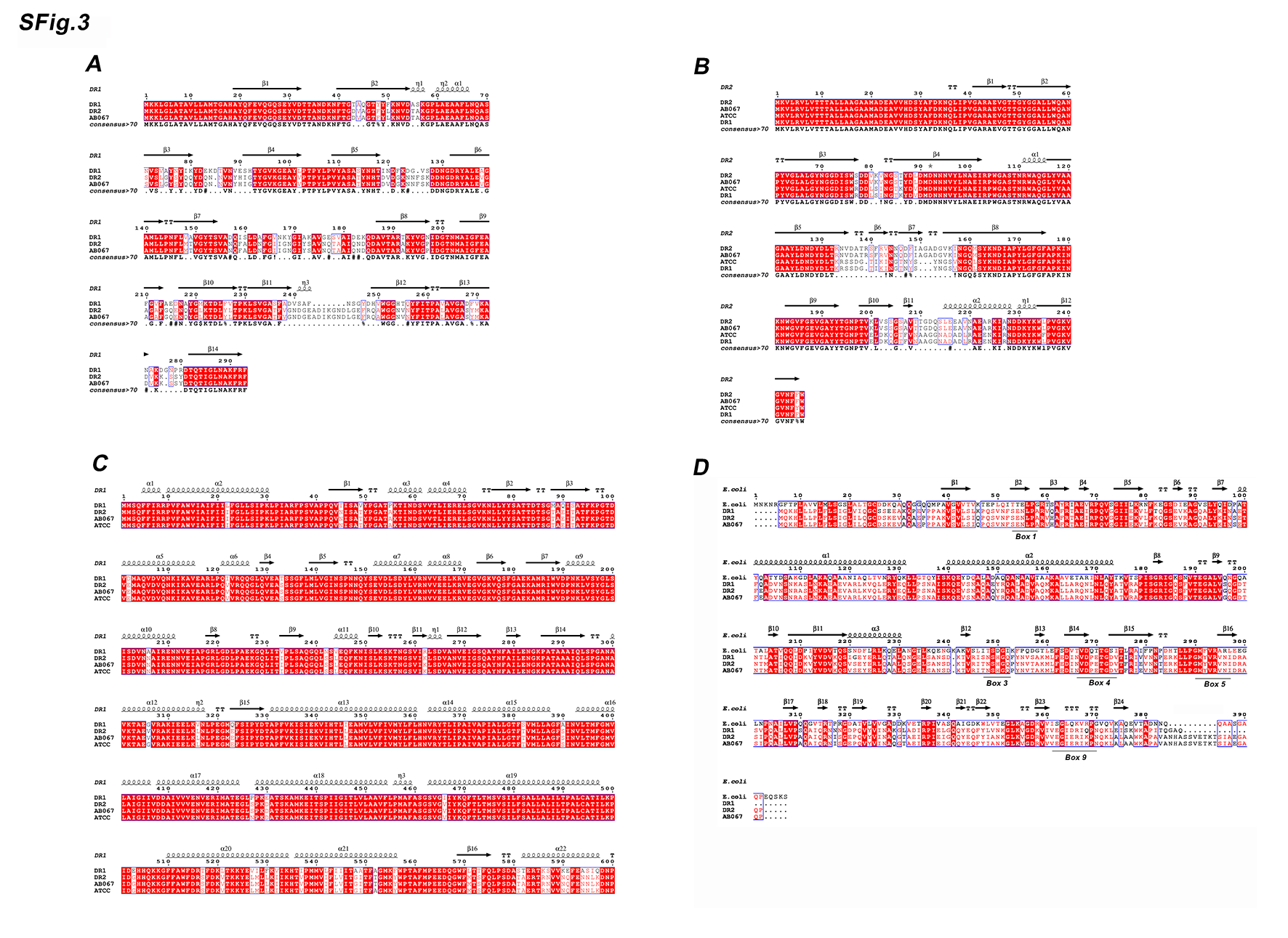

Supplement: Supplementary Figure 3 — Multiple-sequence alignment of resistance determinants from A. baumannii strains. Sequence alignments of homologs of CarO [A, CHQ89_07730 (DR1), CHQ90_00810 (DR2), A8A08_11745 (AB067)], Omp33–36 [B, CHQ89_12430 (DR1), CHQ90_11410 (DR2), A8A08_14050 (AB067)], adeA [C, NOXP01000001 (DR1), NOXO01000021 (DR2) and LZOC01000017 (AB067)], adeB (D) were performed using CLUSTAL Omega (https://www.ebi.ac.uk/Tools/msa/clustalo) and formatted in ESPript server (http://espript.ibcp.fr/ESPript/cgi-bin/ESPript.cgi). The secondary structural elements are shown on each of the alignments using PDB templates of 4fuv.1 for CarO (A), 6gie.1 for Omp33-36 (B), 7kge.1 for AdeB (C) and E. coli AcrA structure (PDB 5o66) for AdeA (D). The mapping of boxes 1–9 in AdeA sequence alignment represents probable interaction boxes as described in McNeil et al. (2019). The arrows indicate a β-sheet, the coils indicate α-helices, TT indicates β turns, and η indicates 310 helices. Residues strictly conserved have a colored background and are indicated by boldface letters; residues conserved between groups are boxed. [file Image_3.tif]

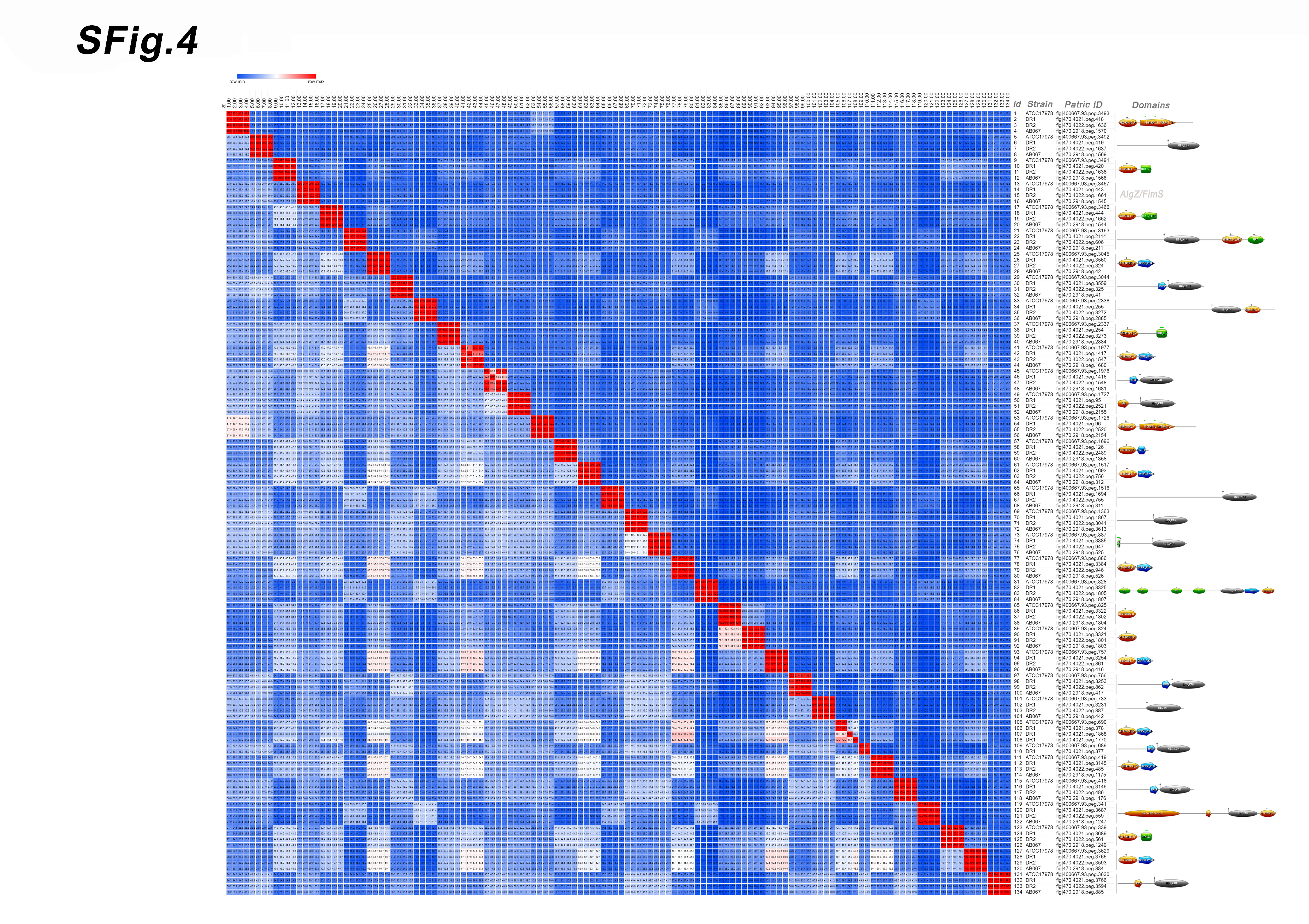

Supplement: Supplementary Figure 4 — The TCS domain organization in A. baumannii. The different signaling proteins found in A. baumannii strains DR1, DR2, and AB067 are depicted in here. The different signaling proteins chosen in A. baumannii strains DR1, DR2, and AB067 are homologs of proteins found in A. baumannii ATCC 17898; namely A1S_0234 Helix-turn-helix, Fis-type, A1S_0235 sensor protein, A1S_0236 BarA-associated response regulator UvrY, A1S_0260 AlgZ/FimS, A1S_0261 positive alginate biosynthesis regulatory protein, A1S_0574 signal transduction histidine-protein kinase BarA, A1S_0748 transcriptional response regulator, OmpR family, A1S_0749 histidine kinase, A1S_1393, A1S_1394 LuxR family, A1S_1753 LuxR family, A1S_1754 histidine kinase, A1S_1977 nitrogen regulation protein NtrB, A1S_1978 NtrC, A1S_2006 response regulator NasT, A1S_2137 DNA-binding response regulator, A1S_2138 histidine kinase, A1S_2287 Sensor protein qseC, A1S_2750 histidine kinase, A1S_2751 LuxR family, A1S_2811 twitching motility protein PilG, A1S_2814 twitching motility protein PilH, A1S_2815 twitching motility protein PilG, A1S_2883 response regulator BaeR, A1S_2884 sensory histidine kinase BaeS, A1S_2906 sensor histidine kinase, A1S_2937 two-component system response regulator QseB, A1S_2938 copper sensory histidine kinase CusS, A1S_3229 response regulator Omp, A1S_3230 osmolarity sensory histidine kinase EnvZ, A1S_3302 sensor histidine kinase, A1S_3304 LuxR family, A1S_3374 regulatory protein PhoB, A1S_3376 phosphate regulon sensor protein PhoR respectively. [file Image_4.TIF]

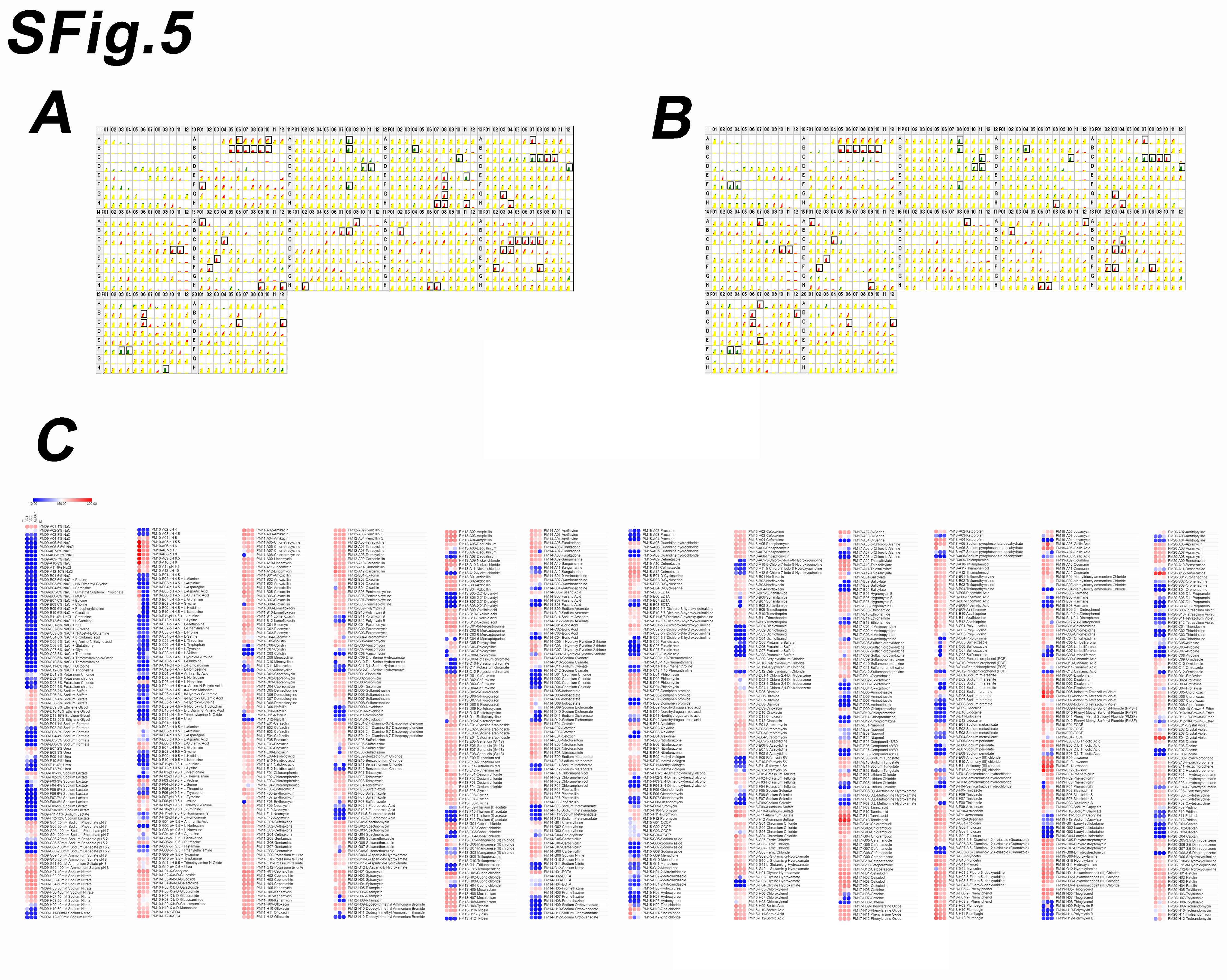

Supplement: Supplementary Figure 5 — Phenotype microarray analysis of A. baumannii DR1, DR2, and AB067 strains. Phenotypic microarray (PM) analysis of A. baumannii DR1, DR2, and AB067 strains were performed to determine the metabolic utilization profiles using PM09 to PM20 plates. Novel Sequence Types in Acinetobacter baumannii kinetic growth response curve for A. baumannii DR1 and DR2 (A) and A. baumannii DR1 and AB067 (B) strains in PM09 to PM20 plates (top panel left as PM09 to right bottom end panel as PM20). The PM data of PM09 to PM20 plates of DR2 (green color) was compared with DR1 (red color), yellow color indicates the equivalent or overlapping growth response of DR1 and DR2 strains. Few wells were enclosed with boxes indicate that the wells were found to be significantly different. The phenotype microarray data of A. baumannii DR1, DR2, and AB067 strains for PM09–PM20 plates were analyzed and depicted in heat map profiles (C). Each dot represents Omnilog values represented all time points of bacterial growth. The Omnilog values were considered for phenotype analysis. The gradient heat maps were generated for A. baumannii DR1, DR2, and AB067 strains for each PM plates from PM09 to PM20 using Omnilog values in Morpheus online tool (https://software.broadinstitute.org/morpheus) which displayed blue color for relative lower growth and red color represents high growth phenotype. [file Image_5.TIF]
